# Printable logic circuits comprising self-assembled protein complexes

**DOI:** 10.1038/s41467-022-30038-8

**Published:** 2022-04-28

**Authors:** Xinkai Qiu, Ryan C. Chiechi

**Affiliations:** 1 Stratingh Institute for Chemistry, University of Groningen, Nijenborgh 4, 9747 AG Groningen, The Netherlands; 2 Department of Chemistry, North Carolina State University, Raleigh, NC 27695-8204 United States; 3 Present Address: Optoelectronics Group, Cavendish Laboratory, University of Cambridge, JJ Thomson Avenue, Cambridge, CB3 0HE UK

**Keywords:** Electronic materials, Molecular self-assembly, Electron transfer, Molecular electronics

## Abstract

This paper describes the fabrication of digital logic circuits comprising resistors and diodes made from protein complexes and wired together using printed liquid metal electrodes. These resistors and diodes exhibit temperature-independent charge-transport over a distance of approximately 10 nm and require no encapsulation or special handling. The function of the protein complexes is determined entirely by self-assembly. When induced to self-assembly into anisotropic monolayers, the collective action of the aligned dipole moments increases the electrical conductivity of the ensemble in one direction and decreases it in the other. When induced to self-assemble into isotropic monolayers, the dipole moments are randomized and the electrical conductivity is approximately equal in both directions. We demonstrate the robustness and utility of these all-protein logic circuits by constructing pulse modulators based on AND and OR logic gates that function nearly identically to simulated circuits. These results show that digital circuits with useful functionality can be derived from readily obtainable biomolecules using simple, straightforward fabrication techniques that exploit molecular self-assembly, realizing one of the primary goals of molecular electronics.

## Abstract (web summary)

Proteins are promising molecular materials for next-generation electronic devices. Here, the authors fabricated printable digital logic circuits comprising resistors and diodes from self-assembled photosystem I complexes that enable pulse modulation.

## Introduction

The ability to wire individual molecules to electrodes has matured into molecular electronics that promise unique functionality beyond what is possible with conventional materials^1^; however, the complexity of incorporating single molecules in nano-junctions precludes technological applications in the near future^2,3^. Devices comprising (self-assembled) molecular ensembles sandwiched between two electrodes, on the other hand, can already be miniaturized and integrated via conventional semiconductor fabrication^4,5^. Many strategies have been developed to realize such molecular ensemble junctions^6^, yet it remains a challenge to combine useful functions^7^, accessible fabrication^8^, and stability in practice^9^. Advances in device fabrication, integration and stability typically yield resistors (e.g., junctions comprising alkanes), while advances in function are demonstrated in test-bed or transient junctions. Proteins are a promising class of molecules with the potential to bridge this divide; they are inherently more robust than small molecules, support coherent tunneling over longer distances^10–12^, exhibit complex functionality^7^ and are easy to work with insofar as they lead to highly reproducible molecular-electronic devices^13^.

Photosystem I (PSI) is a functional nano-device that is produced by and readily isolated from photosynthetic organisms. It is a trimeric membrane protein complex that converts photon energy into separated electron/hole pairs to generate chemical reduction potential as part of natural photosynthesis^14^. Due to the difference in hydrophobicity between the sides and the top/bottom (which is common for membrane proteins), PSI can be induced to self-assemble on surfaces with a preferred orientation^15–19^; in particular, linkers with hydrophilic terminal groups (e.g., hydroxyl group and carboxylic acid) form strong hydrogen bonds with the hydrophilic luminal or stromal surface of PSI trimers, positioning either the P700 reaction center or the F_*B*_ iron–sulfur complex next to the substrate. We have used self-assembled monolayers (SAMs) of thiols on Au and peptides engineered by phage display on tin-doped indium oxide (ITO) to orient PSI with near-perfect selectivity^20^. This high degree of anisotropy translates into the temperature-independent rectification of tunneling current that depends on the absolute orientation of the PSI complexes^21^. We used conductive probe atomic force microscopy (CP-AFM) and eutectic Ga-In^22^ (EGaIn) electrodes to relate the degree and direction of orientation to the magnitude and polarity of rectification empirically and produced soft biophotovoltaic devices based on these assemblies of oriented PSI on Au^23^. There are two limiting factors to that approach: (i) the use of linker-SAMs introduces a tunneling barrier between the PSI and the Au electrode that creates a substantial series resistance, and (ii) the rectification on those junctions is too weak for practical applications.

Here, we eliminate both of the limiting factors by taking advantage of the ability of fullerene (C_60_) derivatives to self-assemble on Au^9^. The fullerene cages are strongly coupled to Au, which facilitates the injection of charges while presenting functional groups that perfectly orient the PSI complexes. Altering these functional groups results in dense SAMs of PSI with either unidirectional or random orientation, leading to robust molecular diodes and resistors, respectively. We incorporated these SAMs into integrated circuits with printed EGaIn electrodes to create molecular logic circuits and pulse modulators composed entirely of self-assembled protein-based circuit elements that operate by activationless long-range charge-transport (e.g., coherent quantum mechanical tunneling).

## Results

### Fabrication of SAMs of PSI

The fabrication of a diode begins with the formation of a SAM of phenyl-C61-butyric acid^24^ (PCBA) by immersing freshly prepared Au (111) on mica (Au^mica^) in a tetrahydrofuran (THF) solution of PCBA for 24 h. Subsequent immersion in an aqueous solution of PSI for 24 h produces a SAM of unidirectionally oriented PSI noncovalently bound to PCBA linkers via strong hydrogen bonding between carboxylic acid and the hydrophilic stromal surface of PSI trimers (see Methods for details of sample preparation, the determination of PSI orientation is discussed below). Replacing the THF/PCBA solution for phenyl-C61-butyric acid methyl ester (PCBM) in chloroform results in a SAM of randomly oriented PSI. (The choice of solvents is based exclusively on the solubility of the fullerene derivatives, e.g., PCBA is sufficiently soluble in THF to form SAMs, but not in chloroform.) Atomic force microscopy (AFM) shows the formation of uniform SAMs from PCBA in THF (Supplementary Fig. 1b) and PCBM in chloroform (Supplementary Fig. 1c) as well as the subsequent SAMs of PSI (Supplementary Fig. 1d, f) formed on top of the fullerene layer. SAMs of PCBA grown from chloroform showed clear signs of aggregation due to its poor solubility (Supplementary Fig. 1a). The formation of the SAMs of PSI on PCBA follows fast kinetics; a dense SAM of PSI forms on the SAMs of PCBA within 0.5 h and remains stable for 24 h, showing almost invariant surface morphology (Supplementary Fig. 2) and surface potential (translated into the work function of Au^mica^ substrate measured by Kelvin probe force microscopy, Supplementary Figs. 3 and 4). We also prepared SAMs of PSI on PTEG-1, which is a fullerene derivative bearing a triethylene glycol chain. Although the SAMs of PTEG-1 were uniform, the PSI complexes tended to aggregate instead of forming dense monolayers(Supplementary Fig. 1e), demonstrating the specificity of the alkyl and carboxylic acid functionalities of PCBM and PCBA. As controls, we formed monolayers of bovine serum albumin (BSA) (Supplementary Fig. 1g) and denatured PSI (Supplementary Fig. 1h) on PCBA; these data are discussed in detail below.

### Charge-transport measurements

**Fig. 1:**
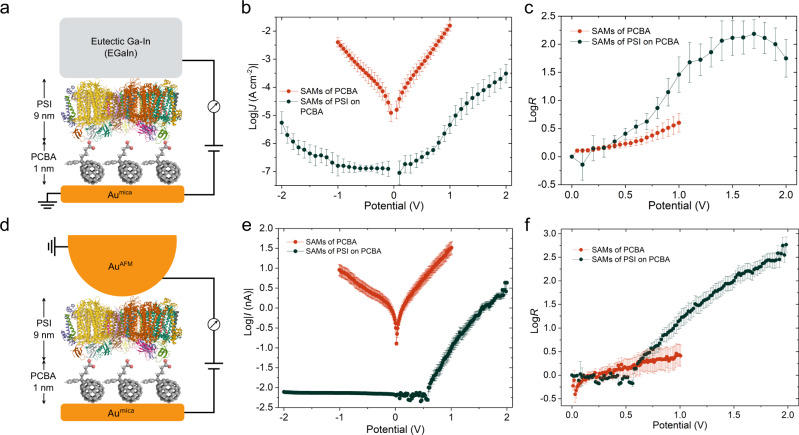
Characterization of the charge-transport properties of the SAMs of PCBA and the SAMs of PSI on PCBA. **a** Schematic of the Au^mica^/PCBA//PSI//EGaIn junctions. **b** Plots of log|*J*| versus potential of Au^mica^/PCBA//EGaIn junctions and Au^mica^/PCBA//PSI//EGaIn junctions. **c** Plots of log*R* versus potential of Au^mica^/PCBA//EGaIn junctions and Au^mica^/PCBA//PSI//EGaIn junctions. **d** Schematic of the Au^mica^/PCBA//PSI//Au^AFM^ junctions. **e** Plots of log|*I*| versus potential of Au^mica^/PCBA//Au^AFM^ junctions and Au^mica^/PCBA//PSI//Au^AFM^ junctions. **f** Plots of log*R* versus potential of Au^mica^/PCBA//Au^AFM^ junctions and Au^mica^/PCBA//PSI//Au^AFM^ junctions. Error bars represent 95% confidence intervals. Drawings of molecules do not correspond to their actual sizes.

We contacted the various combinations of SAMs with conical tips of EGaIn (Fig. 1a) and Au-coated AFM tips (Au^AFM^, Fig. 1d), forming junctions with the structures Au^mica^/linker//EGaIn (or Au^AFM^) and Au^mica^/linker//PSI//EGaIn (or Au^AFM^), where ‘/’ denotes the interfaces defined by chemisorption and ‘//’ by physisorption (the yields are shown in Supplementary Table 1 for CP-AFM junctions and Supplementary Table 2 for EGaIn junctions, respectively). To eliminate mechanical deformation induced by the probe (particularly Au^AFM^; EGaIn, which is a non-Newtonian fluid in ambient conditions, facilitates soft contacts) and its substantial effect on charge-transport characterization^25–27^, we chose a soft cantilever and fixed the force of the Au^AFM^ at the smallest 1.4 nN for all CP-AFM junctions (see Methods for details). The SAMs of PCBA exhibit a significantly higher conductance than the SAMs of PSI on PCBA in both EGaIn and CP-AFM junctions (Fig. 1b,e) due to the exponential dependence of tunneling currents on the distance between the electrodes, confirming that the SAMs of PSI mitigate the flow of current and not defects and/or pinholes. Although some asymmetry is present in the *J/V* and *I/V* curves of the SAMs of PCBA in EGaIn and CP-AFM junctions, the magnitude of rectification is significantly higher for SAMs of PSI, with rectification ratios (*R* = |*J*(+)|/|*J*(-)|) of ≈ 100 (max. ≈ 300) in (large-area) EGaIn junctions and ≈ 575 (max. ≈ 850) in CP-AFM junctions. As is shown schematically in Fig. 1d and Supplementary Fig. 23, CP-AFM junctions comprise individual PSI complexes, 98% of which rectified, indicating near-perfect unidirectional orientation (a detailed discussion on the determination of PSI orientation is provided in Supporting Information)^15,20,21^. This is further corroborated by the enhanced power conversion efficiency and short-circuit current of dye-sensitized solar cells comprising SAMs of PSI on PCBA, in which the optimized orientation of PSI improves the alignment of charge-transport chain to mitigate the recombination of excitons^28^. The voltage dependence of *R* in Fig. 1c, f clearly show that the SAMs of PSI (on top of the SAMs of PCBA) can withstand higher bias (±2 V) and that they rectify significantly more than the SAMs of PCBA alone. This asymmetry is completely absent for the SAMs of PSI on PTEG-1 in EGaIn junctions (Supplementary Fig. 6), which also yield undetectable currents in CP-AFM junctions, likely due to the anti-fouling nature of glycol ether SAMs against proteins^29^. This result again demonstrates the specific role of the alkyl and carboxylic acid groups, i.e., the C_60_ cages of PCBA, PCBM and PTEG-1 only serve to anchor these groups to Au and do not participate directly in the rectification mechanism.

**Fig. 2:**
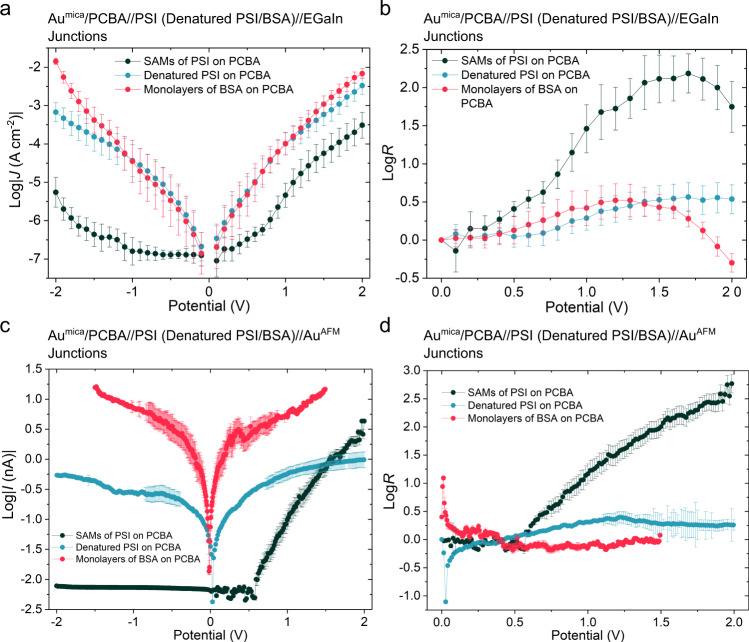
Characterization of the charge-transport properties of the monolayers of different biocomplexes on PCBA. **a** Plots of log|*J*| versus potential of Au^mica^/PCBA//PSI (denatured PSI or BSA)//EGaIn junctions. **b** Plots of log*R* versus potential of Au^mica^/PCBA//PSI (denatured PSI or BSA)//EGaIn junctions. **c** Plots of *I* versus potential of Au^mica^/PCBA//PSI (denatured PSI or BSA)//Au^AFM^ junctions. **d** Plots of log*R* versus potential of Au^mica^/PCBA//PSI (denatured PSI or BSA)//Au^AFM^ junctions. Error bars represent 95% confidence intervals.

To establish the role of intact PSI complexes in the mechanism of rectification, we compared the charge-transport properties of the SAMs of PSI, the monolayers of denatured PSI and BSA on PCBA. Denaturing PSI destroys the electron transport chain and some of the structural rigidity of the complex, while BSA is a structurally intact protein that lacks an electron transport chain or any metal centers. There are no signs of intact PSI trimers or features of similar dimensions in the monolayers of denatured PSI on PCBA (Supplementary Fig. 1h). The monolayers of BSA on PCBA are intact, but less densely packed compared to the SAMs of PSI on PCBA (Supplementary Fig. 1g) despite their smaller sizes, further corroborating the specificity of the interactions between the carboxylic acid groups of PCBA and the periphery of PSI that is absent in BSA. The monolayers of denatured PSI and BSA on PCBA are more conductive than the SAMs of active PSI in both EGaIn junctions and CP-AFM junctions, reflecting the lack of densely packed protein complexes. They also exhibit symmetric *J/V* curves and small rectification ratios (Fig. 2), demonstrating that an intact PSI is required for rectification (the small rectification in the monolayers of BSA on PCBA that lack an electron transport chain could be ascribed to the asymmetric EGaIn junctions in which electrodes have different work functions^30^, in contrast to CP-AFM junctions). While BSA and denatured PSI exhibit similar magnitudes of conductance in EGaIn junctions (Fig. 2a), the former is more conductive than the latter in CP-AFM junctions (Fig. 2c). We ascribe this difference to the lower packing density of denatured PSI; in CP-AFM junctions we contact the denatured complexes individually with the Au^AFM^ tip, while in EGaIn junctions, exposed regions of the underlying SAM of PCBA between the denatured PSI complexes contributes to the current. It is coincidence that the curves overlap in Fig. 2a.

### Mechanism of rectification

The rectification of SAMs of PSI was first observed using thiol-SAMs bearing polar or ionic functional groups to orient the PSI complexes^21^. Earlier work correlated the direction of the electron transport chain in isolated reaction centers to the polarity of rectification^15^, but the polarity in SAMs was reversed. That observation, coupled with the lack of observable thermally activated processes, led to the conclusion that rectification was instead driven by the permanent dipole moments of the alpha helices in the periphery of the complexes. The rectification ratios of the SAMs of PSI oriented by thiol linkers were much lower (*R* < 10, Supplementary Fig. 7) than in this work, and fullerenes themselves have been shown to rectify via a thermally activated tunneling-hopping (non-coherent tunneling) mechanism^31^.

**Fig. 3:**
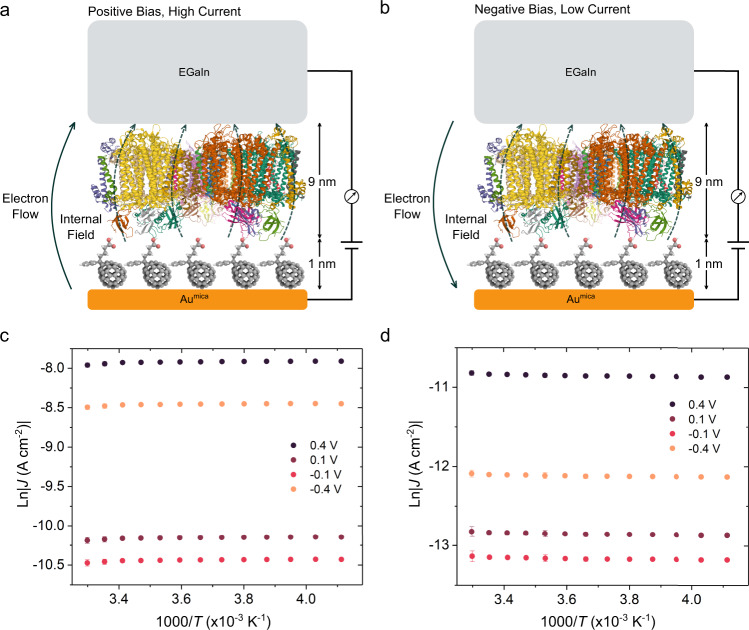
Direction of the internal electric field (dashed lines) that arises from the PSI dipole moment within Au^mica^/PCBA//PSI//EGaIn junctions, which are shown with EGaIn biased both positively and negatively with respect to the normal wiring of EGaIn junctions. The direction of this field goes from negative to positive in the PSI complex. **a** When a positive bias is applied, the direction of the internal electric filed is the same as the applied bias. **b** When a negative bias is applied, the electric field from the applied bias opposes the internal electric field of the PSI complexes. Thus, this mechanism predicts the rectification from the junctions. **c** Plots of ln|*J*| as a function of temperature in Au^mica^/PCBA//EGaIn junctions ranging from 243 K to 303 K measured at an interval of 5 K. **d** Plots of ln|*J*| as a function of temperature in Au^mica^/PCBA//PSI//EGaIn junctions ranging from 243 K to 303 K measured at an interval of 5 K. Drawings of molecules do not correspond to their actual sizes.

To investigate the mechanism of rectification in PCBA/PSI junctions, we fabricated microfluidic devices following literature procedures^9,21^ and acquired *J/V* traces for Au^mica^/PCBA//EGaIn junctions and Au^mica^/PCBA//PSI//EGaIn junctions at different temperatures. Figure 3c, d and Supplementary Fig. 8 show the evolution of *J* from 243 K to 303 K at ±0.1 V and ±0.4 V for Au^mica^/PCBA//EGaIn junctions and Au^mica^/PCBA//PSI//EGaIn junctions, respectively. The magnitudes of *J* and *R* are invariant with temperature for both junctions, showing only a slight decrease in *J* on Au^mica^/PCBA//EGaIn junctions and a slight increase in *J* on Au^mica^/PCBA//PSI//EGaIn junctions near room temperature, both of which are insignificant and do not constitute activation energies. These data establish that the mechanism of charge-transport through the SAMs of PCBA and the SAMs of PSI on PCBA does not involve thermally activated process, e.g., hopping^32,33^. Recent studies on STM-based junctions comprising monolayers of azurin emphasized that mechanical compression on the proteins and/or tip displacement reduces tunneling distance to effect high coherent tunneling probability^27^; we explicitly controlled our experimental parameters to minimized those effects, e.g., by using the uniform, smallest contact force in CP-AFM junctions and soft contact in EGaIn junctions, and the agreement in data obtained from both junctions ruled out the contribution from displaced tip. We calculated the tunneling decay coefficients of PSI and BSA by fitting the currents obtained from junctions comprising the SAMs of PCBA and the SAMs of PSI (or BSA) on PCBA against the thicknesses in the simplified Simmons Equation, *J* = *J*_0_exp(−*βd)*, in which *J*_0_ represents the injection current of an ideal junction with molecules present, *β* represents the tunneling decay coefficient, and *d* represents the width of tunneling barrier. We estimated a *β* of 0.11 Å^−1^ for PSI and 0.14 Å^−1^ for BSA, in which the former is similar to literature values (0.08 Å^−1^–0.16 Å^−1^ on thiol-based linkers)^21^, and the latter is lower despite the different substrates (0.27 Å^−1^ from monolayers of BSA on SiO_2_/Si substrate)^30^. The efficient tunneling across proteins, identified by the low *β*, can be ascribed to temperature-independent sequential tunneling in which the presence of metal centers in proteins facilitate the vibronic coupling of the charge carrier with the vibrational modes of the molecules^34^; however, it does not explain the origin of rectification.

We have previously observed vacuum level shifts induced by molecular dipoles in SAMs^35^ that affect tunneling charge-transport^36^ enough to induce rectification^37^. Recent studies on charge-transport through SAMs of oligopeptides suggests that interactions among high-energy occupied orbitals in consecutive amide bonds facilitate efficient coherent charge-transport by superexchange tunneling, and the built-in dipole of the ensemble can give rise to rectification^38,39^. Very small changes in *J* with temperature have been reported in SAMs where asymmetric charge-transport was mediated by activationless resonant tunneling induced by the Stark effect^40^. Similarly, we observed a slight, symmetric change in *J* in Fig. 3c and d that we ascribe to the thermal expansion of the liquid EGaIn electrode^41^. The characterization on charge-transport across the SAMs of PSI on PCBA did not yield any temperature-dependence, but there is a strong dependence of rectification on the director SAMs, which—as we have reported previously^21^—is indicative of rectification driven by the collective action of built-in permanent dipoles (and orientation) of the alpha helices encircling the reaction center of the PSI complexe and is not related to charge-transfer through the electron transport chain or PCBA. This observation is further corroborated by the analysis on the statistical moments in which the skewness and kurtosis^42^ of the current distribution on the SAMs of PSI on PCBA exhibited bias-dependence as a result of the alignment between the built-in dipoles and the external electric field (see Supporting Information for detailed discussion). This is a striking result, since the combined thicknesses of the fullerene layer and PSI complexes is approximately 10 nm (the PSI trimer is 9 nm thick and the PCBA is 1 nm thick, the determination of thicknesses is discussed in Supporting Information), about a factor of three higher than multi-heme cytochromes (MHCs)^10^. As illustrated in Fig. 3a, b and Supplementary Fig. 9, the built-in electric field in PSI trimers is directed from the F_*B*_ subunit (higher redox potential) to the P700 electron donor (lower ground-state redox potential). In the SAMs of PSI on PCBA, the F_*B*_ subunit is positioned next to the PCBA linker and the P700 electron donor is adjacent to the EGaIn electrode. The direction of the external field is the same as the built-in electric field at positive bias (Fig. 3a) but opposes the built-in electric field at negative bias (Fig. 3b), therefore giving rise to a lower tunneling probability of electrons in the latter. To further support this hypothesis, we compared the rectification of the SAMs of PSI on PCBA fabricated on Au substrates with varied roughness. The built-in electric field of PSI on rough Au films evaporated onto Si wafer (Au^Si^, roughness ≈1.7 nm, Supplementary Fig. 10a) does not align with the external field as well as on smooth Au^mica^ (roughness ≈0.5 nm, Supplementary Fig. 10b), leading to rectification ratios a factor of 10 smaller on the former compared to the latter (Supplementary Fig. 10b, c). Thus, the degree of rectification is directly related to the orientation of the (dipoles within) PSI complexes, but efficient charge-transport is independent of orientation, suggesting that asymmetric charge-transport through PSI is a collective property of the alpha helices that surround the reaction center; to the extent that individual peptides or polar groups affect the electrical characteristics, it is insignificant^43–45^. Our observations, therefore, fully support a mechanism of rectification that is driven by the built-in electric field of PSI complexes and demonstrate that useful magnitudes of rectification can be achieved with PSI trimers with optimal orientation. And, since the polarity of rectification opposes the redox gradient of the electron transport chain (Supplementary Fig. 9), we do not believe that metal centers (e.g., Mg and Mn atoms in the reaction center of PSI) participate in the tunneling process as they do in MHCs^10,46^, suggesting that long-range charge-transport is a property of folded polypeptides and not metalloproteins. Our experimental results and survey of theoretical models (see Supporting Information for detailed discussion) suggest long-range coherent tunneling as a possible mechanism for the efficient charge-transport across the SAMs of PSI; however, we cannot rule out activation-less Marcus–Landauer hopping^47^. Although we are confident in our assertions regarding the origin of rectification, more rigorous experimental investigation is needed to establish the mechanism of charge-transport.

### Fabrication of printable logic circuits

**Fig. 4:**
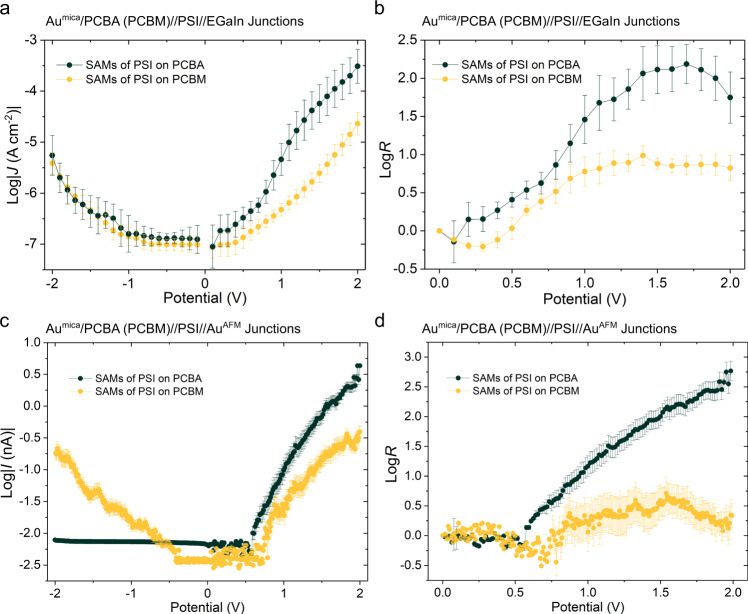
Characterization of the charge-transport properties of the SAMs of PSI on different linkers. **a** Plots of log|*J*| versus potential of Au^mica^/PCBA (PCBM)//PSI//EGaIn junctions. **b** Plots of log*R* versus potential of Au^mica^/PCBA (PCBM)//PSI//EGaIn junctions. **c** Plots of log|*I*| versus potential of Au^mica^/PCBA (PCBM)//PSI//Au^AFM^ junctions. **d** Plots of log*R* versus potential of Au^mica^/PCBA (PCBM)//PSI//Au^AFM^ junctions. Error bars represent 95% confidence intervals.

A useful feature of molecular electronics is the ability to integrate circuit elements into individual molecules^48,49^. But the correlation between orientation and function in SAMs of PSI and the ability to print EGaIn^50^ create the possibility for integrated circuits composed entirely of PSI, i.e., multiple junctions wired together without any conventional circuit elements such as resistors or diodes. To create resistors from PSI, we replaced PCBA with PCBM, a fullerene derivative functionalized with methyl ester instead of carboxylic acid, presenting a surface that is neither protic nor ionic; the intermediate polarity of SAMs of PCBM does not strongly favor one orientation of PSI. Figure 4a, c show the resulting nearly symmetric *J/V* characteristics, which translate into much smaller values of *R*, particularly in CP-AFM junctions (Fig. 4b, d). Note that the *I/V* and *R/V* curves from CP-AFM junctions are averages from randomly sampled complexes (see Methods for details). While the asymmetry of the electrodes makes perfectly symmetric *J/V* curves nigh impossible, junctions comprising SAMs of PSI on PCBM are functionally resistors across the ±2 V window shown in Fig. 4.

In previous studies, the polarity of rectification depended on the orientation of the PSI complexes, which was controlled by the identity of the underlying SAM on which the PSI complexes self-assembled^20,21^; however, the magnitude of *R* was too small to extract useful function. The magnitude of *R* for the SAMs of PSI oriented by PCBA, on the other hand, is sufficiently high to construct diode logic circuits^51^. Transistor-based logic circuits displaced diode-based logic circuits in consumer electronics because field-effect transistors switch faster than conventional diodes, which are limited by capacitive charging in PN diodes. Molecular rectifiers, however, operate in the tunneling regime and demonstrably switch at 17 GHz, with an extrapolated cut-off frequency of 520 GHz^52^. Thus, while molecular transistors are certainly of interest^53^, they do not preclude other approaches—the first molecular logic devices were based on arrays of two-terminal logic gates^54,55^. Logic circuits based on molecular diodes are, therefore, both a demonstration of the capabilities of molecular ensemble junctions and a potentially useful technological application.

**Fig. 5:**
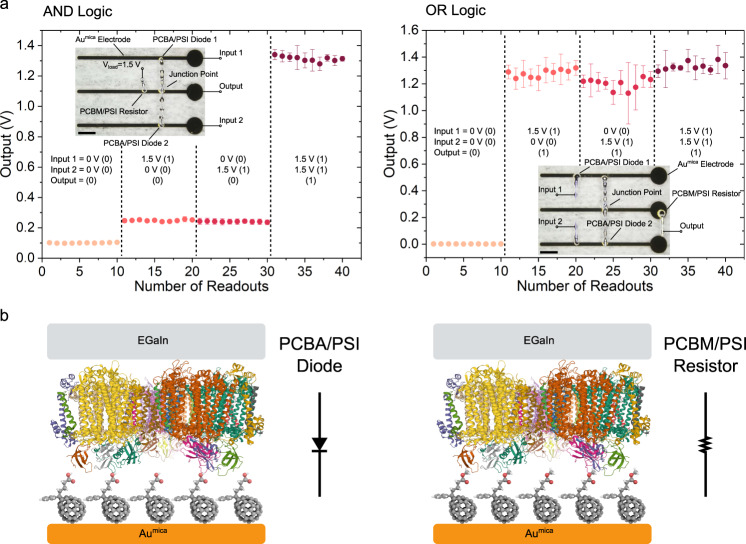
Characterization of the functions of logic circuits comprising Au^mica^/PCBA//PSI//EGaIn junctions and Au^mica^/PCBM//PSI//EGaIn junctions and printed EGaIn electrodes. **a** Schematics (inset) and plots of output voltage of AND logic circuits (top) versus varied input under a constant load of 1.5 V and OR logic circuits (bottom) versus varied input. Numbers in brackets represent the input and output values in a truth table. Error bars represent standard deviation of the mean. Scale bars of the insets are 1.5 mm. **b** Schematics of the diode (left) comprising a Au^mica^/PCBA//PSI//EGaIn junction and the resistor (right) comprising a Au^mica^/PCBM//PSI//EGaIn junction used in the logic circuits.

We constructed AND and OR logic gates, each composed of two PCBA/PSI diodes (i.e., tunneling junctions comprising SAMs of PSI on PCBA) and one PCBM/PSI resistor (i.e., a tunneling junction comprising a SAM of PSI on PCBM). The circuit diagrams and optical micrographs of the EGaIn wiring are shown in Supplementary Fig. 11. A diode AND circuit works by placing a forward bias across a resistor and two shunt diodes in series with the output. A reverse bias on both diodes is required to block the shunting, leading to a high voltage on the output. A diode OR circuit reverses the polarity of the diodes and places the resistor between the output and ground, such that a high voltage on either input puts a forward bias across one of the diodes, leading to a high voltage on the output. Thus, both of them are three-terminal logic circuits with diodes at the inputs. To form the diodes and resistors of these circuits, we fabricated the SAMs of PSI on PCBA on the two outermost Au^mica^ strips, and the SAMs of PSI on PCBM on the center strip such that contacting the former with EGaIn produces a diode and the latter a resistor, as shown in Fig. 5a (see Methods for details). To form a logic circuit, we printed EGaIn directly onto the SAM-supporting Au^mica^ strips, using EGaIn both to form the diodes and resistors (Fig. 5b) and to wire them together, thus forming all-protein diode logic in one printing step. The performance of both logic gates is exemplary; as shown in Fig. 5a, the output of the AND gate is below 300 mV (which is assigned to FALSE or 0 in the truth table) unless both inputs are raised to 1.5 V, raising the output to 1.3 V (which is assigned to TRUE or 1 in the truth table). Likewise, the output of the OR gate is 1.3 V (TRUE/1) if either or both inputs are raised to 1.5 V, and ≈0 V (FALSE/0) if both inputs are kept at 0 V.

The outputs of the AND and OR gates are stable (in time and voltage) and are functionally distinct from each other (Supplementary Fig. 12). This stability is derived from the remarkable robustness of the junctions; the ability to preserve stable, reproducible charge-transport properties under the electrostatic pressure under high voltage and the mechanical stress of EGaIn printing is unique to the SAMs of PSI formed on fullerenes (Supplementary Figs. 16 and 17), the average Young’s modulus of which is 1.5 GPa (Supplementary Fig. 5). Thiol-SAMs of ferrocenes (e.g., ferrocenyl undecanethiol) and alkanes (e.g., hexadecane) function as molecular rectifiers and resistors using conical tips of EGaIn as electrodes, with yields of working junctions close to 90% (Supplementary Figs. 18 and 19); however, the yields in printed EGaIn junctions drop to zero due to their mechanical fragility. The yield of the considerably more robust SAMs of PSI, by contrast, is close to 90% (Supplementary Fig. 19).

**Fig. 6:**
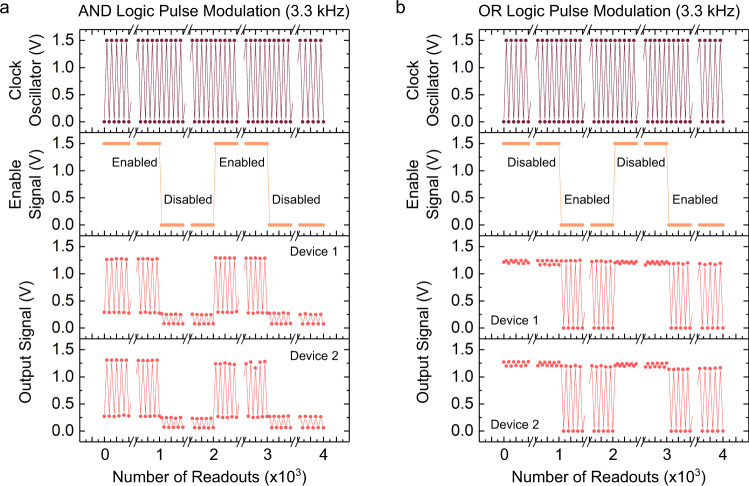
Fast pulse modulations on logic circuits comprising Au^mica^/PCBA//PSI//EGaIn junctions and Au^mica^/PCBM//PSI//EGaIn junctions and printed EGaIn electrodes. **a** Operations of pulse modulation achieved by two AND logic circuits at a frequency of 3.3 kHz. **b** Operations of pulse modulation achieved by two OR logic circuits at a frequency of 3.3 kHz. Here, the clock oscillator generates a rectangular pulse of 1.5 V at a frequency of 3.3 kHz, the enable signal is switched between 1.5 V and 0 V to modulate the output of the circuit. Note that the connecting lines between data points do not correspond to the actual waveform.

To demonstrate the usefulness of all-protein AND and OR gates, we implemented them in pulse modulators. Figure 6 shows, from top to bottom, plots of voltage of a clock oscillator, an enable signal and the output signals of two AND (Fig. 6a) and OR (Fig. 6b) gates comprising PSI with the clock oscillator and the enable signal wired to their inputs, versus the number of readouts. The clock oscillator was set to generate a 1.5 V rectangular pulse at 3.3 kHz (3.3 kHz is the highest frequency accessible on the source meter used in this work). When wired to one input of an AND gate, the pulses of the clock oscillator are transmitted to the output only if the voltage of the enable signal on the other input is high (i.e., it enables the pulse transmission by an AND operation); when the enable signal is switched off, the pulses of the clock oscillator are damped and not readable at the output. The modulation using an OR gate is the opposite; when the enable signal is high, the output is high regardless of the clock oscillator, thus the oscillation is only observed at the output when the enable signal is low. These outputs are in excellent agreement with simulations of pulse modulators shown in Supplementary Figs. 20 and 21. Further investigation on the frequency dependence of PCBA/PSI diodes showed stable *J/V* curves (Supplementary Fig. 13a) and *R* values (Supplementary Fig. 13b) from 1 Hz to 3.3 kHz. Both AND and OR logic gates behave indifferently at a frequency of 3.3 kHz (Fig. 5 and Supplementary Fig. 15) in comparison to those at a low frequency (e.g., 0.5 Hz in Supplementary Fig. 14), retaining the capability of pulse modulation over 4000 readouts.

## Discussion

The field of molecular electronics presents an intriguing opportunity for interdisciplinary research on molecule-scale devices; however, the putative goal is the development of electronic devices that enhance, supplant and/or extend the functionality of classical semiconductors. To accomplish this goal, three major problems must be solved: (i) the instability of molecules attached to surfaces/sandwiched between electrodes, (ii) the transient nature of metal–molecule–metal junctions, and (iii) large junction-to-junction variability in electrical outputs. We addressed all these problems by replacing chemically unstable anchors (most commonly thiols) with strong, reversible, chemically inert fullerene-metal interactions; directly printing liquid metal electrodes and interconnects onto pre-patterned electrodes bearing self-assembled monolayers; and replacing fragile small molecules with nanoscopic protein complexes that derive their functionality from simple electrostatic interactions. The resulting devices reproducibly function as exemplary AND and OR logic gates in ambient conditions, without any encapsulation or special handling. While more work is needed to further miniaturize and integrate these devices to explore high-frequency operation and more complex function, we have proven the concept of integrated circuit elements derived from a single protein complex, the function of which is controlled entirely by self-assembly, and demonstrated the utility of this approach by constructing logic gates that function as pulse modulators.

## Methods

### Preparation of monolayers of PCBA and monolayers of PSI on PCBA

Self-assembled monolayers (SAMs) of PCBA were prepared by incubating fresh Au (111) epitaxially grown on mica in a saturated solution of PCBA in THF (1 mg in 2.4 mL, the solution was filtered before use) for 24 h, rinsed with THF and dried under a gentle flow of N_2_. SAMs of PCBM was prepared similarly by incubating the substrate in a chloroform solution of PCBM (0.5 µM, the solution was filtered before use). SAMs of PSI on PCBA were prepared by incubating the freshly fabricated SAMs of PCBA in an aqueous solution of PSI (1.04 µM) for 24 h, rinsed with demineralized water and dried under a gentle flow of N_2_. Monolayers of denatured PSI and BSA on PCBA were prepared similarly. PCBA and PSI were obtained from our existing laboratory stocks that were used in previous studies. Their preparation is described in Refs. ^24^ and^23^, respectively.

SAMs of PCBA and PCBM as well as SAMs of PSI on PCBA and PCBM used in logic circuits were prepared by continuously flowing solutions in cofabricated parallel microfluidic channels^56^ over thermally deposited Au^mica^ microelectrodes at the same time. The microfluidic channels were prepared by curing polydimethylsiloxane (PDMS, SYLGARD 184, Dow Inc.) over master patterns of the channels on a silicon wafer at 60 °C for 3 h. SAMs of PSI on PCBA were fabricated by first injecting the saturated solution of PCBA in THF (1 µg in 2.4 mL, the solution was filtered before use) into two microfluidic channels at a rate of 0.2 mL h^−1^ for 3 h, rinsed with THF at the same rate, dried with N_2_ and then injecting the aqueous solution of PSI (1.04 µM) at a rate of 0.2 mL h^−1^ for 3 h. SAMs of PSI on PCBM were fabricated by first injecting a chloroform solution of PCBM (0.5 µM, the solution was filtered before use) into one microfluidic channel at a rate of 0.2 mL h^−1^ for 3 h, rinsed with chloroform at the same rate, dried with N_2_ and then injecting the aqueous solution of PSI (1.04 µM) at a rate of 0.2 mL h^−1^ for 3 h. The microfluidic channels were removed and re-applied between drying with N_2_ and the samples were rinsed with demineralized water after the removal of microfluidic channels and dried under a gentle flow of N_2_.

### Atomic force microscopy

PeakForce Tapping AFM, Kelvin probe force microscopy (KPFM), conductive probe AFM (CP-AFM) and PeakForce Quantitative Nanomechanical AFM (PFQNM) were performed on a Bruker AFM Multimode 8 MMAFM-2 equipped with a PeakForce TUNA application module (Bruker). All samples were characterized by AFM. PeakForce Tapping AFM was performed with a ScanAsyst-Air probe (resonant frequency 70 kHz, spring constant 0.4 N m^−1^, Bruker) to characterize the surface morphology of the samples at a scan rate of 0.6 Hz and 800 samples per line. The data were analyzed with Nanoscope Analysis 1.5 provided by Bruker.

KPFM measurements were performed in amplitude modulated mode. Pt/Ir coated conductive probes (SCM-PIT-V2, spring constant 3 N m^−1^, resonant frequency 75 kHz, Bruker) were calibrated on a freshly cleaved highly oriented pyrolytic graphite (HOPG) before the measurements. All samples were scanned at a rate of 0.5 Hz with scan sizes of 2 × 2 µm^2^ and 5 × 5 µm^2^, and 640 samples per line over three different regions. The potential shifts were calculated by fitting the raw data into Gaussian distribution and were later translated into work functions based on the calibrated work function of the probe.

In CP-AFM measurements, samples fabricated on Au^mica^ substrates were contacted with a Au-coated silicon nitride tip with a nominal radius of 30 nm (NPG-10, Bruker, tip A: resonant frequency 65 kHz, spring constant: 0.35 N m^−1^) in TUNA mode with a force of 1.4 nN. The AFM tip was grounded and the sample was biased from −2 V to 2 V and from 2 V to −2 V to record the *I/V* curves (512 data points per trace were taken) for each junction by positioning the tip to each biocomplex. After every junction, the tip was withdrawn and moved to a different spot, and engaged again for all three samples analyzed. Between different samples a new tip was used. The data were analyzed with the same software used for EGaIn using the current *I* instead of the current density *J*. Although the tip is grounded in CP-AFM junctions instead of the substrate, as is done for EGaIn junctions, we previously demonstrated that CP-AFM junctions with normal and reversed wiring simply yield *I/V* curves that mirror each other^21^. Therefore, we mirrored the *I/V* and *R/V* curves of CP-AFM junctions in plots in which we compared CP-AFM and EGaIn junctions to preserve the correct polarity of rectification as determined by the PSI complexes. We chose this approach over performing the measurements with the wiring reversed due to limitations of our CP-AFM setup that preclude reversing the wiring.

Measurements of Young’s modulus were performed in PFQNM mode. The samples were contacted with a Si_3_N_4_ tip with a nominal radius of 2 nm (ScanAsyst-Air, Bruker, resonant frequency 70 kHz, spring constant 0.4 N m^−1^). The deflection sensitivity, spring constant of the cantilever and radius were calibrated both before and after measurements. Deformation and adhesion of the samples were measured under a force load ranging from 50 pN to 2 nN by contacting each PSI complexes in point-and-shoot mode, and later used to calculate the Young’s modulus using the DMT model in Nanoscope Analysis (Bruker).

### EGaIn measurements

Electrical measurements with EGaIn, as well as sample preparation and handling, were performed under ambient conditions. In the measurement, the sample was grounded and the EGaIn was biased. At least three samples were examined for monolayers/bilayers. The potential windows include the following: (1) 0 V → 1 V → 1 V → 0 V, steps of 0.05 V; (2) 0 V → 2 V → 2 V → 0 V, steps of 0.1 V. A total of five trace/retrace cycles were recorded for each junction, and shorts occurred during the measurement (short upon contact with a bias of 1 V or during the cycle) were counted as failure of the junction. Data were processed using the free, open-source GaussFit software (https://github.com/rchiechi/GaussFit).

The variable-temperature measurements were performed on a custom-built cryogenic probe station in vacuum (pressures varied from 6 × 10^−7^ mbar to 3 × 10^−6^ mbar). The devices were slowly cooled down and their *J/V* characteristics were measured from 303 K to 243 K. We biased the EGaIn top electrodes and grounded the Au^mica^ bottom electrode. Using the area defined by the stencil mask and PDMS channels, we measured *J* as a function of *V* at an intervals of 5 K, allowing the devices to stabilize before performing each scan. We fabricated three devices for each type of system (SAMs of PCBA and SAMs of PSI on PCBA) and measured 1 of each at a time. Each scan of a junction was recorded individually and the aggregate dataset was treated identically to the room temperature data as described above. We biased from −0.4 V to +0.4 V at steps of 0.05 V with a delay of 0.1 s. Because the Au strips on mica cannot be prepared by template-stripping (a conventional methodology of creating flat substrates for cross-bar junctions^57^; mica is delaminated upon the application of adhesive to form an insulating layer on Au after template-stripping), the substrates were left with 200-nm-tall steps at both sides of the Au strips on which neither PSI nor PCBA can form densely packed SAMs, causing the junctions to short at high bias. The results of the cross-bar junctions at low bias are consistent with the junctions of conical EGaIn tips and CP-AFM, in which PSI still constitutes a tunneling barrier and facilitates asymmetric charge-transport.

EGaIn electrodes used in logic circuits were fabricated by injecting EGaIn droplets onto the SAMs of PSI on PCBA and PCBM through a customized setup at controlled conditions (EFD, Ultumus V, tip inner diameter 0.10 mm, outer diameter 0.24 mm, injection time 0.04 s, pressure 30.0 kPa and vacuum 1.30 kPa). Interconnections and external contacts were fabricated using the same method. Inputs (e.g., fixed bias and pulses) were generated from two separate source meters (Keithley 2400 and Keithley 6430) and outputs were read out through a multimeter (Fluke, model 233).

## Supplementary information

Supplementary Information

## Data Availability

The raw data used to prepare the EGaIn, CP-AFM and logic circuits as well as the extended analyses in the Supplementary Information have been deposited in the RCCLab Dataverse: 10.5281/zenodo.6419138.

## Code availability

All of the raw data acquired using EGaIn and CP-AFM were processed using Scientific Python. The source code is publicly available at https://github.com/rchiechi/ GaussFit and at this permalink: 10.5281/zenodo.6422417.
