# Supplementary material for: Printable logic circuits comprising self-assembled protein complexes
Source: Nat Commun. 2022 Apr 28;13:2312. doi: 10.1038/s41467-022-30038-8 (PMC9050843; doi:10.1038/s41467-022-30038-8)
Supplement: Supplementary file 1 — Supplementary Information [file 41467_2022_30038_MOESM1_ESM.pdf]

Supplementary Information for: Printable Logic Circuits Comprising Self-assembled Protein Complexes

Xinkai Qiu*[1,2] and Ryan C. Chiechi*[1,3]

[1]Stratingh Institute for Chemistry, University of Groningen, Nijenborgh 4, 9747 AG Groningen, the Netherlands
[2]Current address: Optoelectronics Group, Cavendish Laboratory, University of Cambridge, JJ Thomson Avenue, Cambridge CB3 0HE, United Kingdom
[3]Department of Chemistry, North Carolina State University, Raleigh, North Carolina 27695-8204, United States
*e-mail: xq237@cam.ac.uk
*e-mail: ryan.chiechi@ncsu.edu

Contents

1 Materials and synthesis **S-3**

2 Atomic force microscopy, AFM **S-4**

3 EGaIn measurements **S-7**

4 Simulation of logic circuits **S-17**

5 Determination of thicknesses and PSI orientation **S-20**

6 Possible models for charge-transport mechanisms **S-22**

7 Analysis on skewness and kurtosis **S-27**

1 Materials and synthesis

The synthesis of PTEG-1 and PCBA are described elsewhere.[1,2] 1-(3-(methoxycarbonyl)propyl) – 1-phenyl [6.6]C_{61} was obtained from Solenn B.V. Et_2O as solvent was an analytical-grade reagent and was used as received. Other chemicals used in the synthesis were obtained from Sigma-Aldrich and TCI and used as received. The Ag^{TS} substrates used in this work were made by mechanic template stripping as described else where;[3] we deposited 100 nm Ag (99.99 %) by thermal vacuum deposition onto a 3-inch wafer (without an adhesion layer). Using the UV-curable optical adhesive (OA) Norland 61, we glued 1 cm^2 glass chips on the metal surfaces. The Au^{mica} substrates used in this work were made by thermally depositing 200 nm Au (99.99 %) in vacuum onto mica substrates at an annealing temperature of 350 °C. The substrates were gradually heated up to the annealing temperature over 1 h and kept at that temperature for 19 h until deposition; then the substrates were kept at the annealing temperature for another 2 h and allowed to gradually cooled down to room temperature. All substrates were used immediately after preparation.

2 Atomic force microscopy, AFM

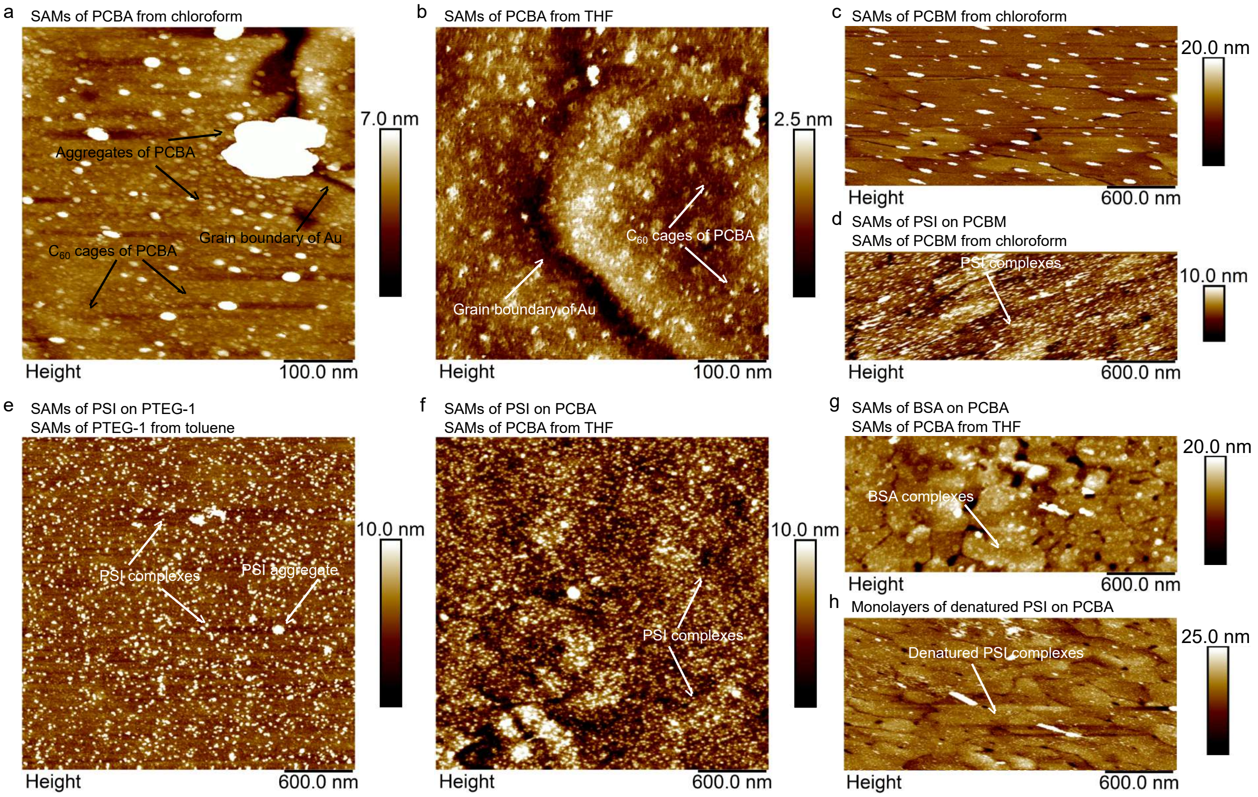

Supplementary Figure 1. Characterization of the morphology of the samples investigated in this work by AFM. a, Height image of the SAMs of PCBA on Au^{mica} grown from a chloroform solution of PCBA. b, Height image of the SAMs of PCBA on Au^{mica} grown from a THF solution of PCBA. c, Height image of the SAMs of PCBM on Au^{mica} grown from a chloroform solution of PCBM. d, Height image of the SAMs of PSI on PCBM on Au^{mica}. The SAMs of PCBM was formed in a chloroform solution of PCBM. e, Height image of the SAMs of PSI on PTEG-1 on Au^{mica}. The SAMs of PTEG-1 was formed in a toluene solution of PTEG-1. f, Height image of the SAMs of PSI on PCBA on Au^{mica}. The SAMs of PCBA was formed in a THF solution of PCBA. g, Height image of the monolayers of bovine serum albumin on PCBA on Au^{mica}. The SAMs of PCBA was formed in a THF solution of PCBA. h, Height image of the monolayers of denatured PSI on PCBA on Au^{mica}. The SAMs of PCBA was formed in a THF solution of PCBA.

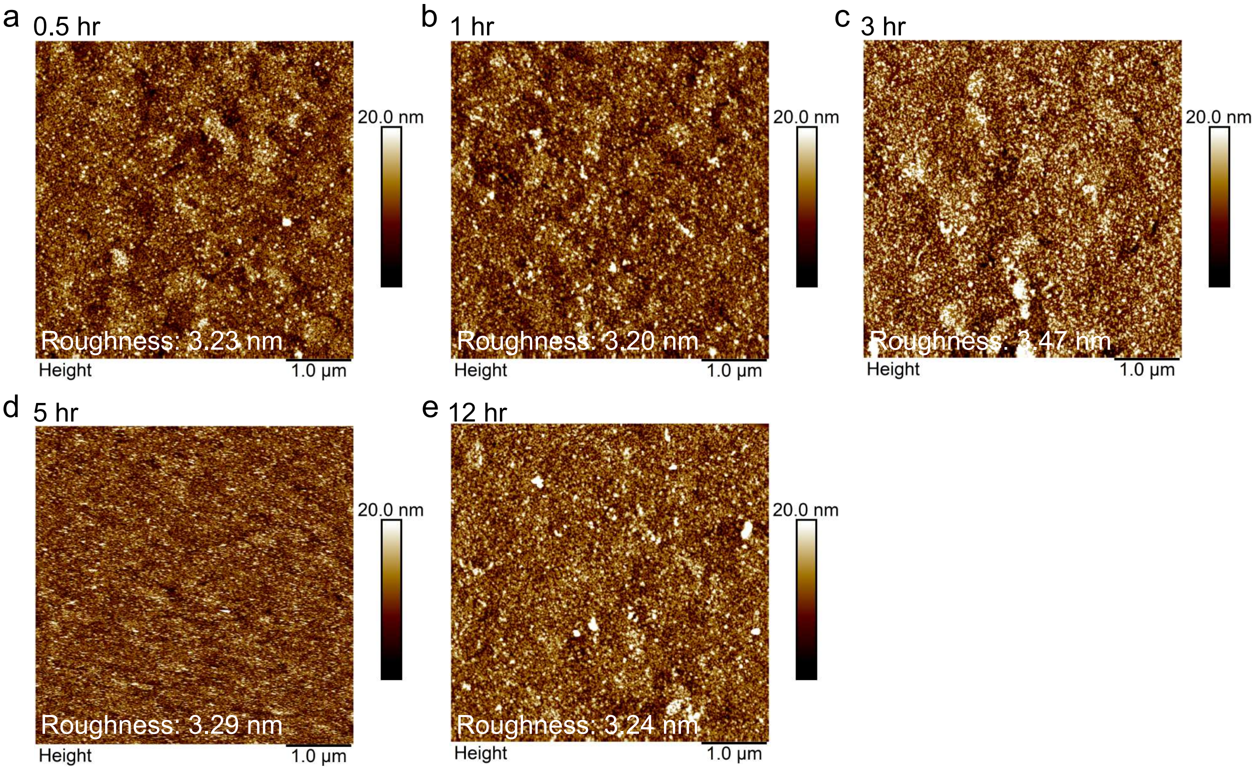

Supplementary Figure 2. The surface morphology and roughness of the SAMs of PSI on PCBA on Au^{mica} after incubating the SAMs of PCBA in the solution of PSI for 0.5 h (a), 1 h (b), 3 h (c), 5 h (d) and 12 h (e).

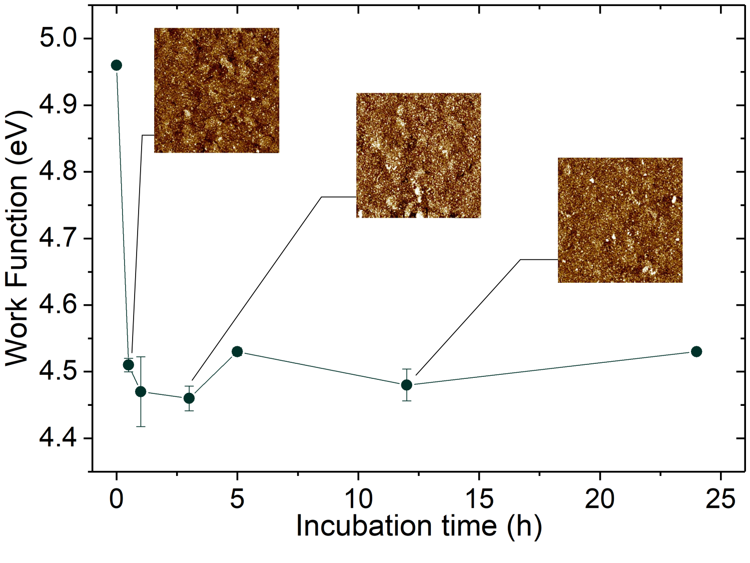

Supplementary Figure 3. The evolution of work function of the Au^{mica} substrate over 24 h by incubating the SAMs of PCBA on Au^{mica} in the solution of PSI. Error bars represent standard deviations.

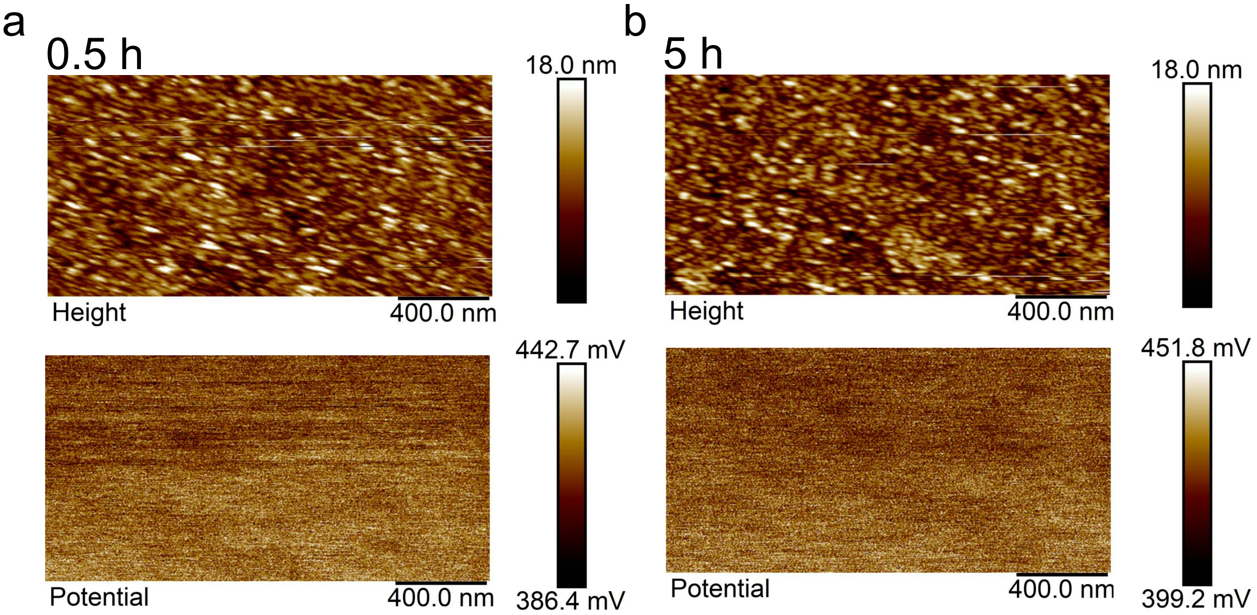

Supplementary Figure 4. The characterization of the surface potential of the SAMs of PSI on PCBA on Au^{mica} using Kelvin probe force microscopy (KPFM). a, Surface morphology (top) and surface potential (bottom, compared to the work function of the Pt/Ir AFM probe) in the same region of the SAMs formed after 0.5 h incubation in the solution of PSI. b, Surface morphology (top) and surface potential (bottom, compared to the work function of the Pt/Ir AFM probe) in the same region of the SAMs formed after 5 h incubation in the solution of PSI.

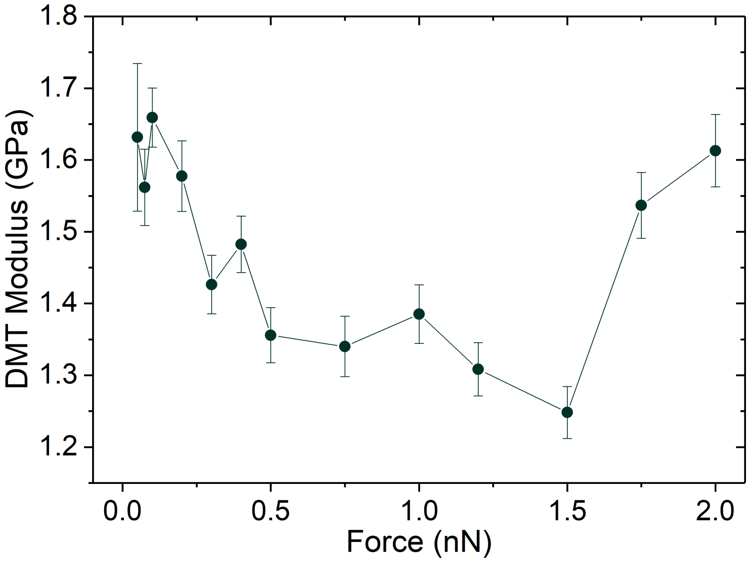

Supplementary Figure 5. Young's moduli of the SAMs of PSI on PCBA on Au^{mica} calculated by Derjaguin-Muller-Toporov (DMT) model under varied force loads applied from an AFM tip. Error bars represent standard deviations.

Table 1: Statistics of CP-AFM junctions of the samples studied in this work.

	Junctions	Shorts	Traces	Yield (%)
**SAMs of PCBA**	394	0	394	100
**SAMs of PSI on PCBA**	203	0	203	100
**SAMs of PSI on PCBM**	223	0	223	100
**Monolayers of denatured PSI on PCBA**	284	0	284	100
**Monolayers of BSA on PCBA**	8	0	8	100

3 EGaIn measurements

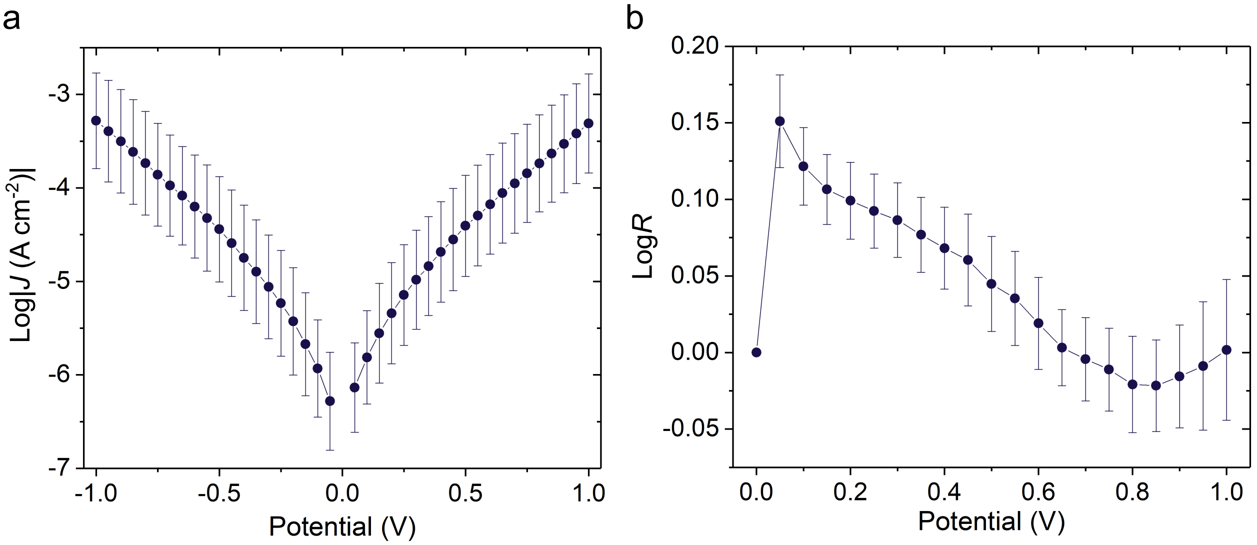

Supplementary Figure 6. Characterization of the charge-transport properties of the monolayers of PSI on the SAMs of PTEG-1 linkers. a, Plots of log $|J|$versus potential of Au^{mica}/PTEG-1//PSI//EGaIn junctions. b, Plots of log R versus potential of Au^{mica}/PTEG-1//PSI//EGaIn junctions. Error bars represent 95% confidence intervals.

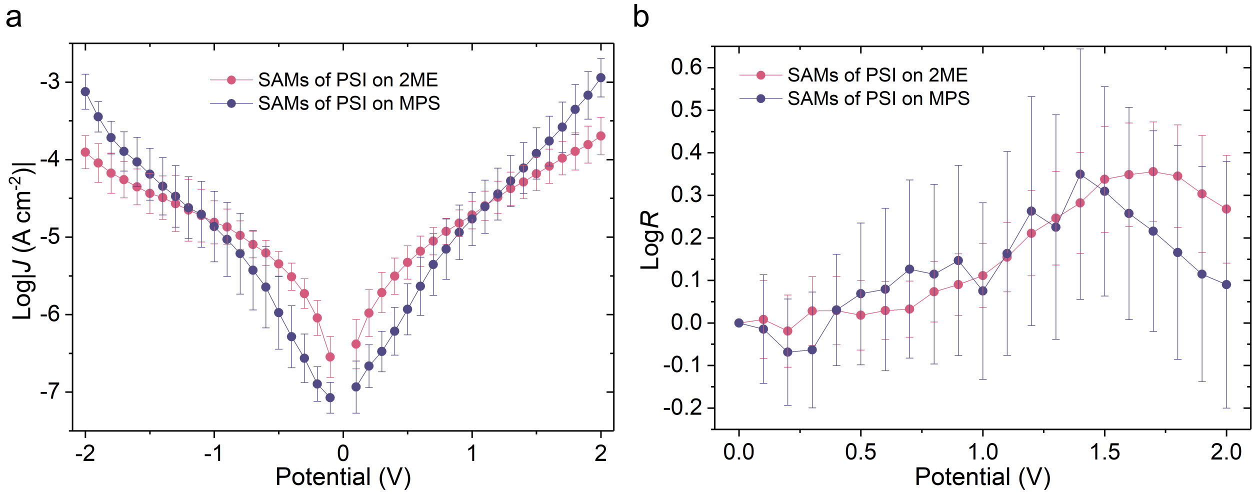

Supplementary Figure 7. Characterization of the charge transport properties of SAMs of PSI on 2ME and MPS linkers. a, Plots of log |*J*| versus potential of Au^{mica}/2ME//PSI//EGaIn junctions and Au^{mica}/MPS//PSI//EGaIn junctions. b, Plots of log*R* versus potential of Au^{mica}/2ME//PSI//EGaIn junctions and Au^{mica}/MPS//PSI//EGaIn junctions. Error bars represent 95% confidence intervals.

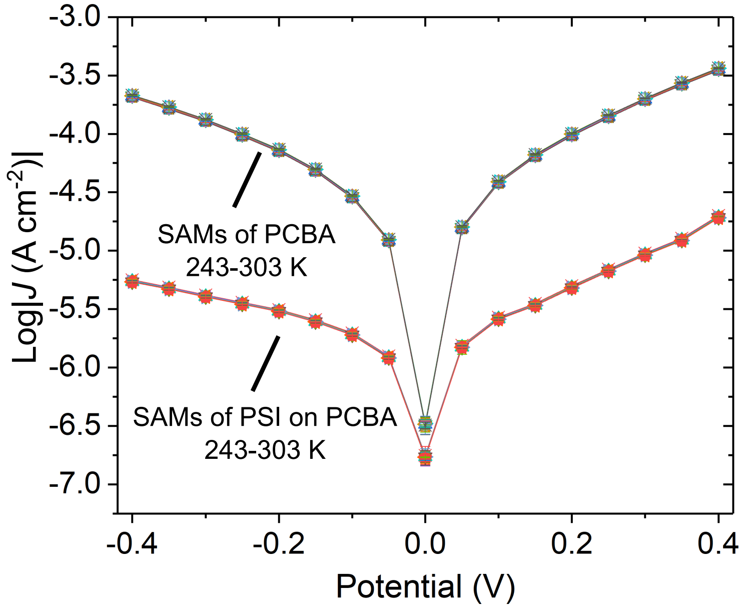

Supplementary Figure 8. Plots of log |*J*| versus potential Au^{mica}/PCBA//EGaIn junctions and Au^{mica}/PCBA//PSI//EGaIn junctions measured at 243-303 K with an interval of 5 K. Error bars represent standard deviations.

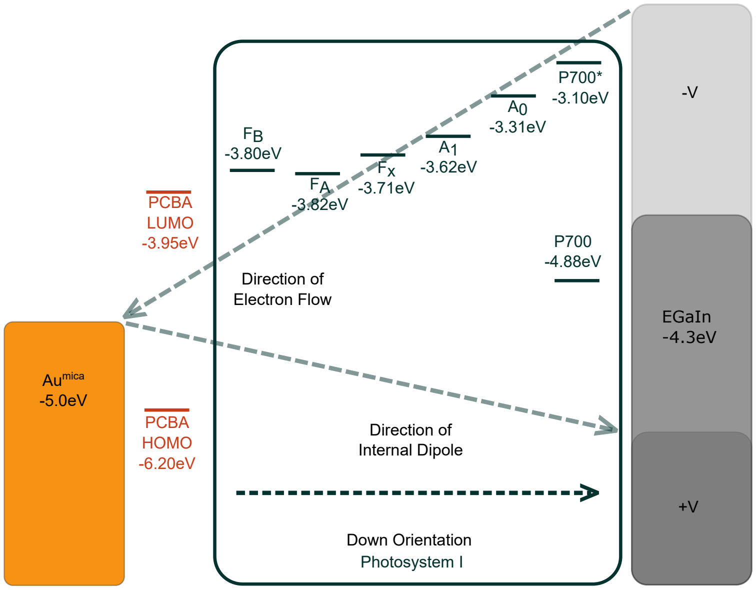

Supplementary Figure 9. Energy level diagram across Au^{mica}/PCBA//PSI//EGaIn junctions. The barrier width is defined by the thickness of one oriented PSI complex, which is depicted in the down-orientation with respect to the natural direction of electron flow. The polarity of rectification is opposite of energy gradient of the electron transport chain, consistent with the mechanism of rectification established in Ref. 4

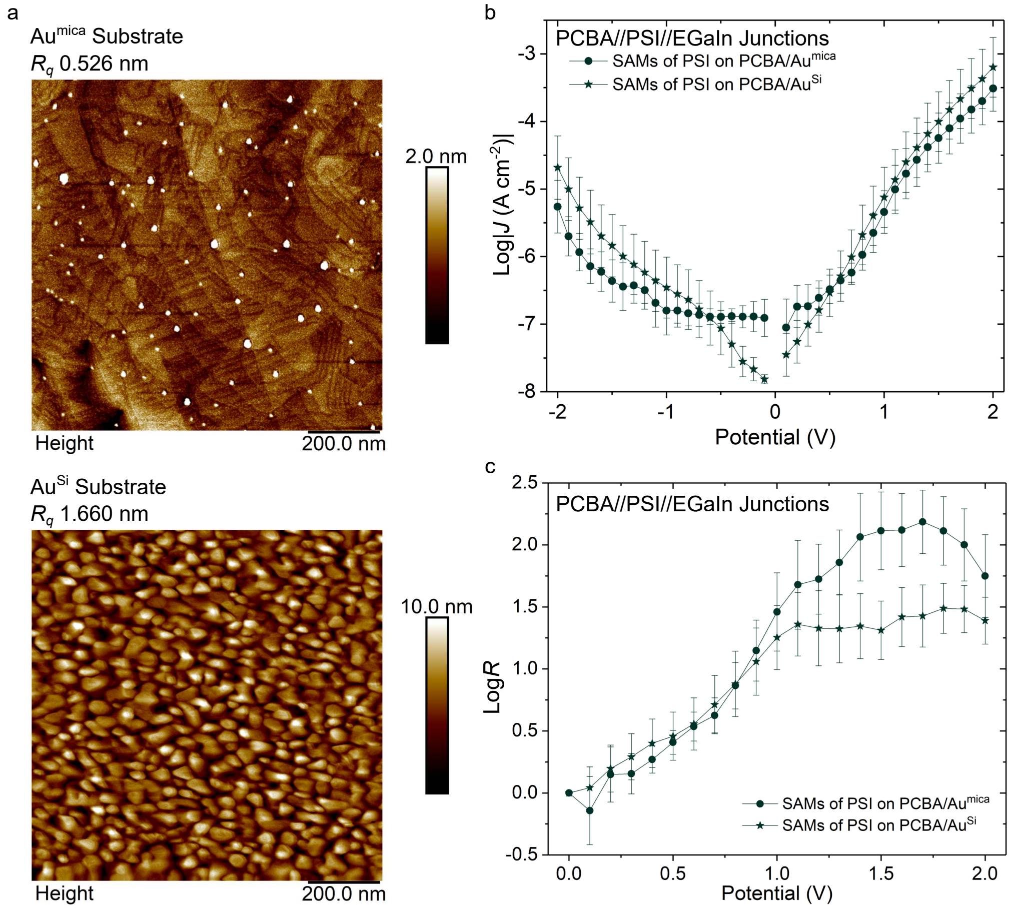

Supplementary Figure 10. Charge-transport properties of the SAMs of PSI on PCBA grown on substrates of different roughnesses. a, Surface morphology of Au^{mica} substrates (top, root mean square roughness 0.526 nm) and Au^{Si} substrates (bottom, root mean square roughness 1.660 nm). The bright spots on the Au^{mica} substrate correspond to the dust particles physisorbed onto the surface from the ambient environment during the fabrication of substrates, which were displaced during the fabrication of SAMs, as shown in Figure S1f and Figure S2. b, Plots of log $|J|$versus potentials of Au^{mica} (or Au^{Si})/PCBA//PSI//EGaIn junctions. c, Plots of log$R$ versus potential of Au^{mica} (or Au^{Si})/PCBA//PSI//EGaIn junctions. Error bars represent 95% confidence intervals.

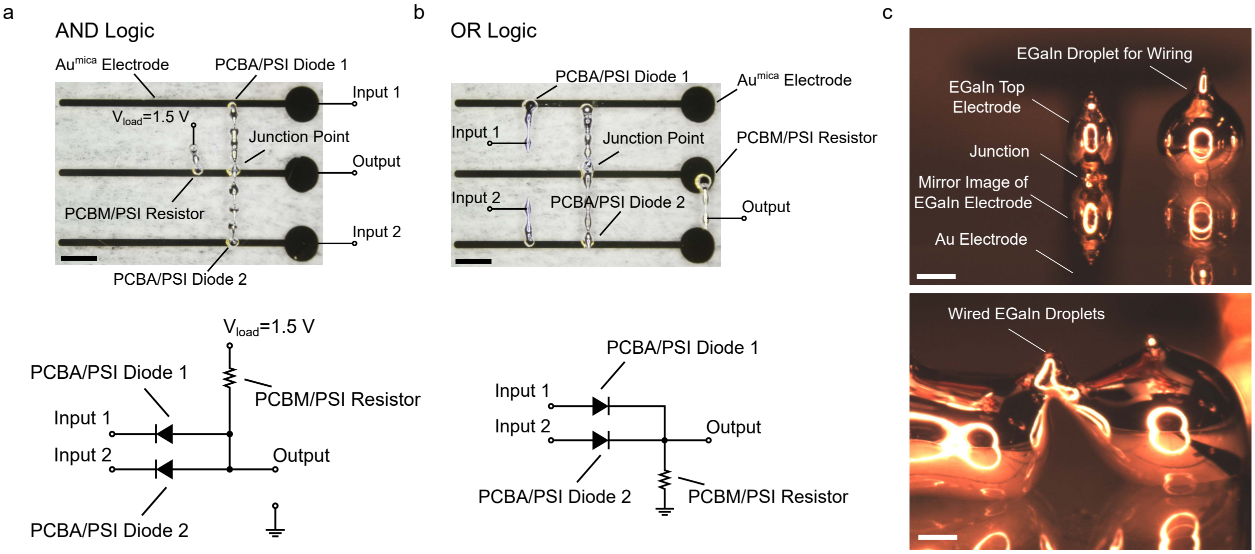

Supplementary Figure 11. Optical micrographs of the logic circuits and their details. a, Optical micrograph (top) and schematic drawing (bottom) of the AND logic circuits comprising PSI complexes with the assignment of electronic components. Scale bar is 1.5 mm. b, Optical micrograph (top) and the schematic drawing (bottom) of the OR logic circuits comprising PSI complexes with the assignment of electronic components. Scale bar is 1.5 mm. c, Optical micrographs showing the size of a junction (approx. 8000 μm^2, top) and the wiring of EGaIn electrodes (bottom). Scale bars are 0.2 mm.

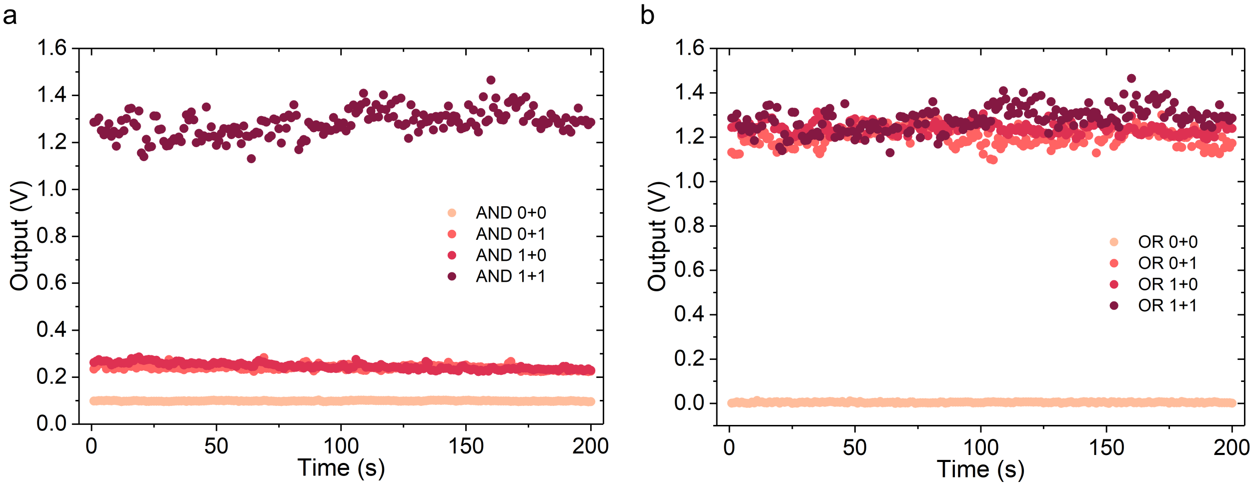

Supplementary Figure 12. Characterization of the stability of AND (a) and OR (b) logic circuits comprising Au^{mica}/PCBA//PSI//EGaIn junctions and Au^{mica}/PCBM//PSI//EGaIn junctions and printed EGaIn electrodes over 200 s. An output voltage was read out every second for each operation.

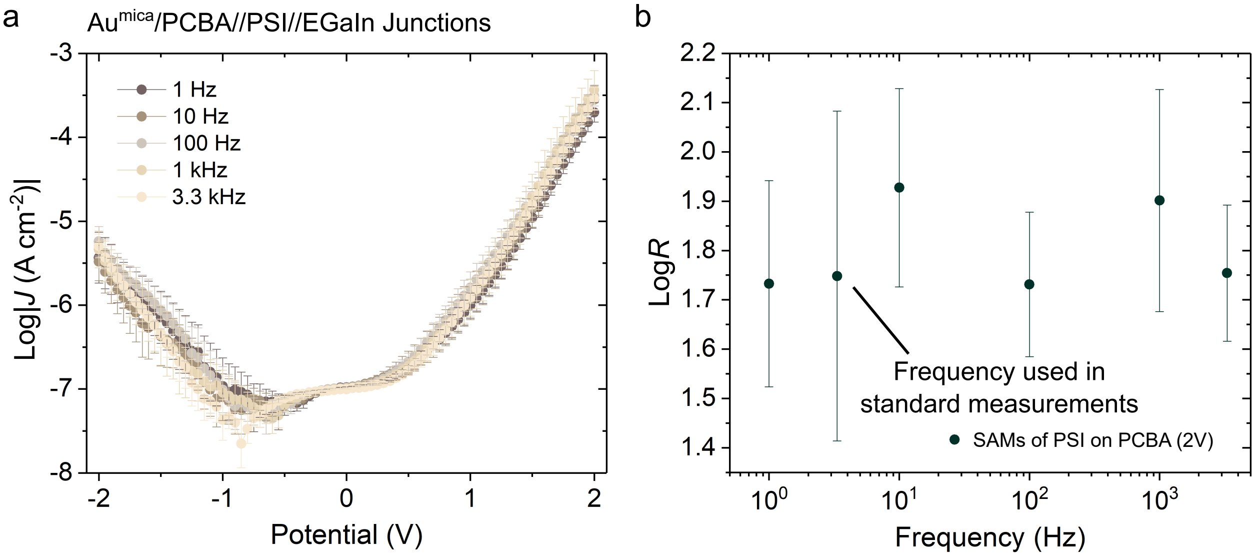

Supplementary Figure 13. Characterization of the charge-transport properties of Au^{mica}/PCBA//PSI//EGaIn junctions and their logic circuits under varied frequencies. a, Plots of log|J|versus potential of Au^{mica}/PCBA//PSI//EGaIn junctions measured between 1-3.3 KHz. b, Plots of log$R$ versus potential of Au^{mica}/PCBA//PSI//EGaIn junctions. Error bars represent 95% confidence intervals.

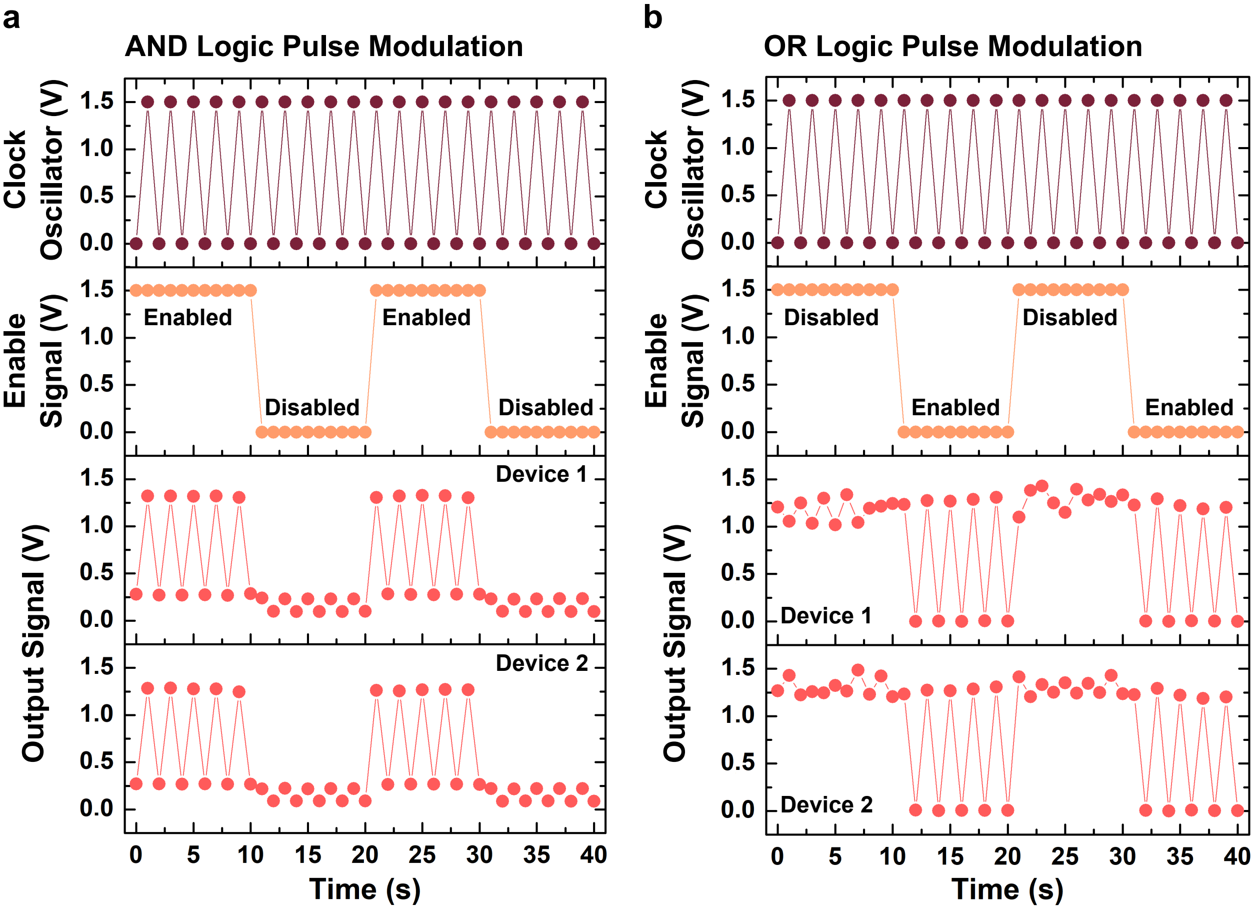

Supplementary Figure 14. Slow pulse modulations on logic circuits comprising Au^{mica}/PCBA//PSI//EGaIn junctions and Au^{mica}/PCBM//PSI//EGaIn junctions and printed EGaIn electrodes. a, Operations of pulse modulation achieved by two AND logic circuits at a frequency of 0.5 Hz. b, Operations of pulse modulation achieved by two OR logic circuits at a frequency of0.5 Hz.

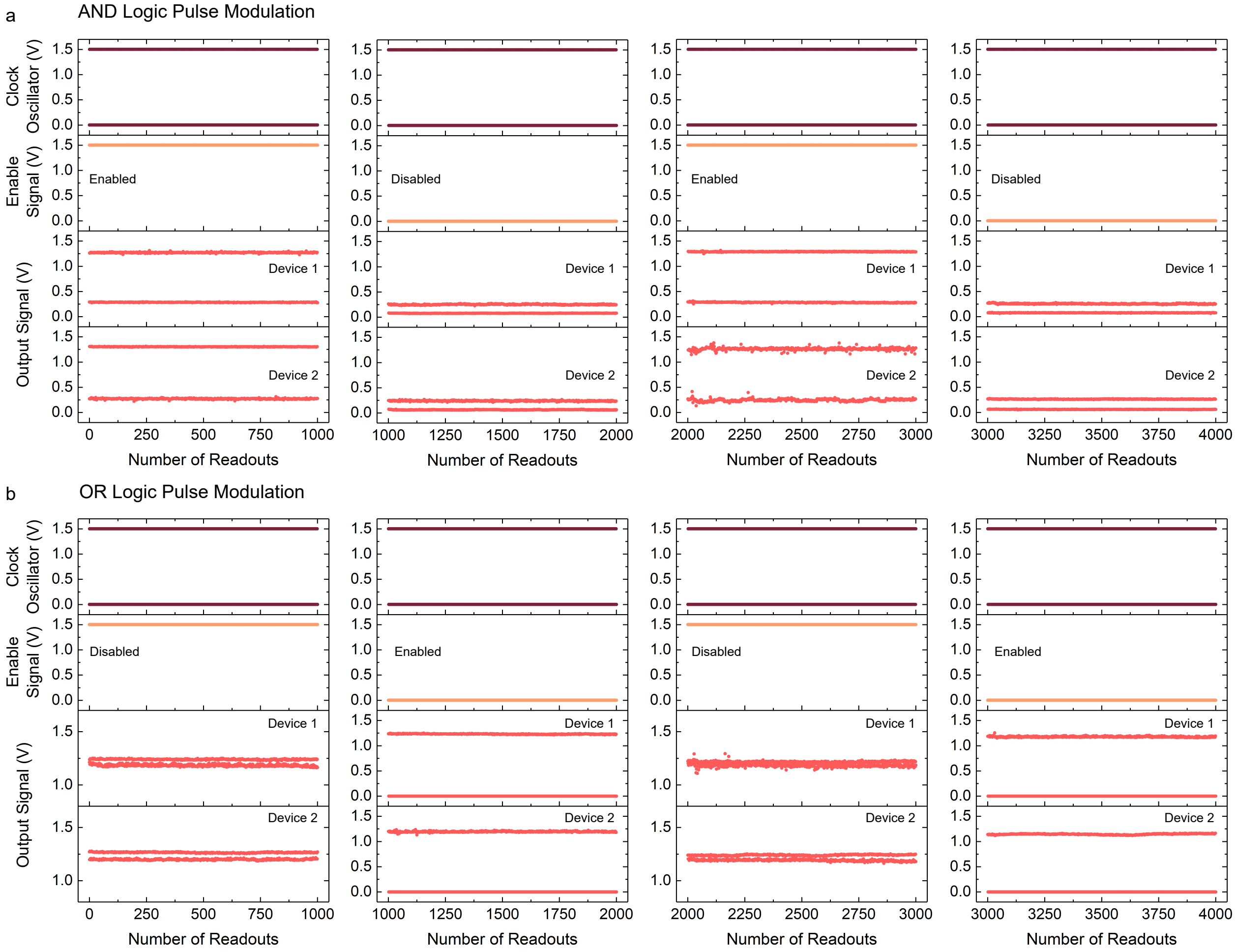

Supplementary Figure 15. Characterization of the charge-transport properties of the printed logic circuits over 4000 readouts. a, Operations of pulse modulation achieved by two AND logic circuits at a frequency of 3.3 kHz. b, Operations of pulse modulation achieved by two OR logic circuits at a frequency of 3.3 kHz. Here, the clock oscillator generates a rectangular pulse of 1.5 V at a frequency of 3.3 kHz, the enable signal is switched between 1.5 V and 0 V to modulate the output of the circuit.

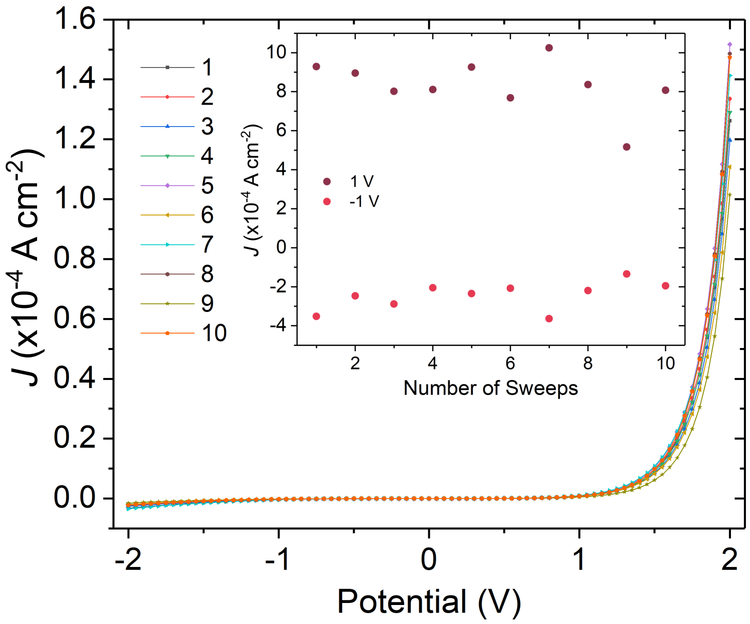

Supplementary Figure 16. Plots of $\boldsymbol{J}$ versus potential from 10 continuous sweeps at a frequency of 3.3 kHz in a Au^{mica}/PCBA//PSI//EGaIn junction. The inset shows the evolution of $\boldsymbol{J}$ measured at -1 V and 1 V over 10 sweeps.

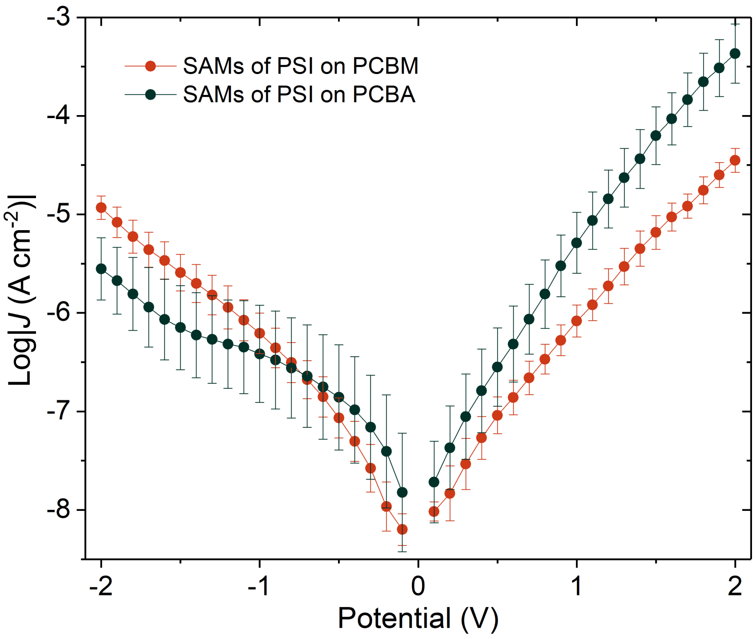

Supplementary Figure 17. Plots of log $|J|$versus potentials of Au^{mica}/PCBA//PSI//EGaIn and Au^{mica}/PCBM//PSI//EGaIn junctions, in which EGaIn electrodes are directly printed onto the SAMs. Error bars represent 95% confidence intervals.

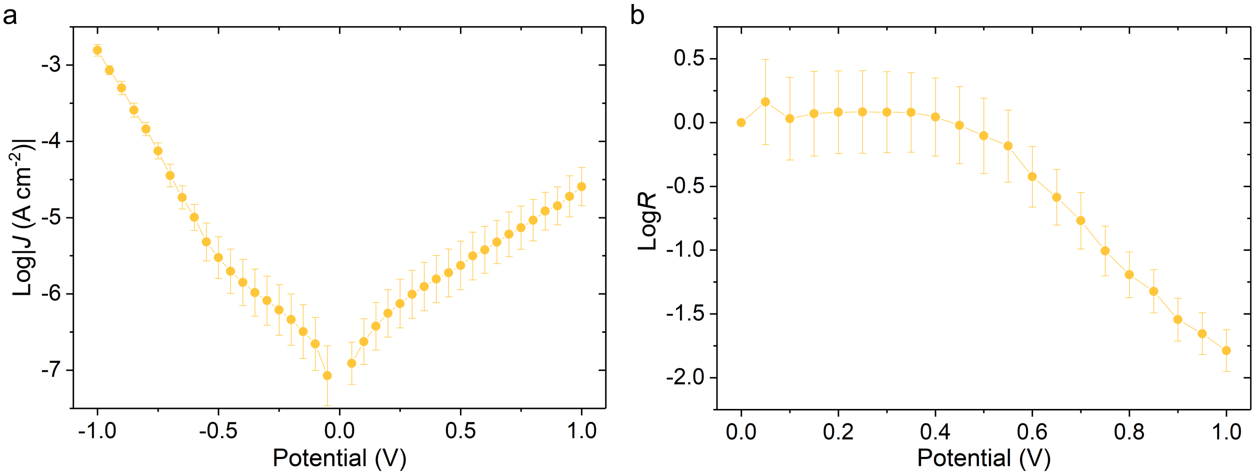

Supplementary Figure 18. Characterization of the charge transport properties of the SAMs of ferrocenyl undecanethiol (FcC11SH) on Ag^{TS}. a, Plot of log$|J|$versus potential for Ag^{TS}/FcC11SH//EGaIn junctions. b, Plot of log$R$ versus potential of Ag^{TS}/FcC11SH//EGaIn junctions. Error bars represent 95% confidence intervals.

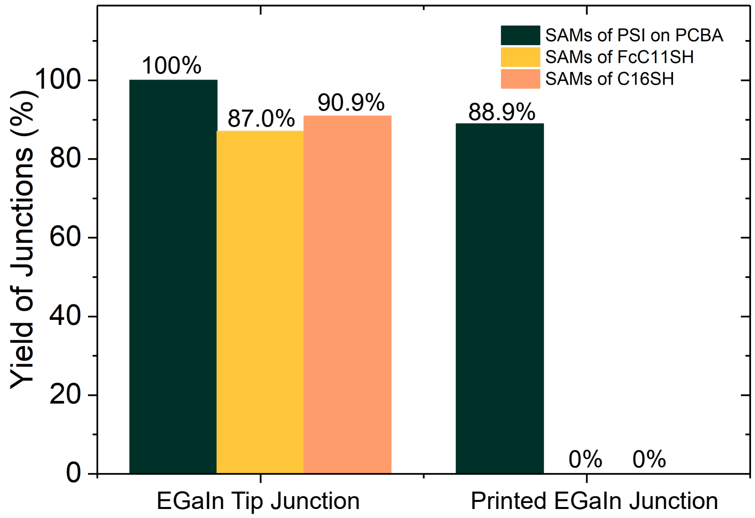

Supplementary Figure 19. Comparison over the yield of working junctions formed by EGaIn conical tip and printed EGaIn electrode.

Table 2: Statistics of EGaIn junctions comprising the samples studied in this work.

	Junctions	Shorts	Traces	Yield (%)
**SAMs of PCBA**	35	6	350	82.9
**SAMs of PSI on PCBA**	25	0	250	100
**SAMs of PSI on PCBM**	40	0	400	100
**Monolayers of denatured PSI on PCBA**	33	3	330	90.9
**Monolayers of BSA on PCBA**	31	6	310	80.6
**SAMs of FcC11SH**	23	3	230	87.0

4 Simulation of logic circuits

The simulation of the functions of logic circuits demonstrated in this work was carried out using an open-source electronic circuit simulator CircuitJS1. The specifications of each simulated electrical component are reproduced from their actual experimental performance. Diodes are set with a saturation current of 3 pA, a series resistance of 1.5 GΩ, an emission coefficient of 2. Resistors are set with a resistance of 47.4 GΩ. Clock oscillators are set with a maximum voltage of 1.5 V in the waveform of pulse with a frequency of 3.3 kHz.

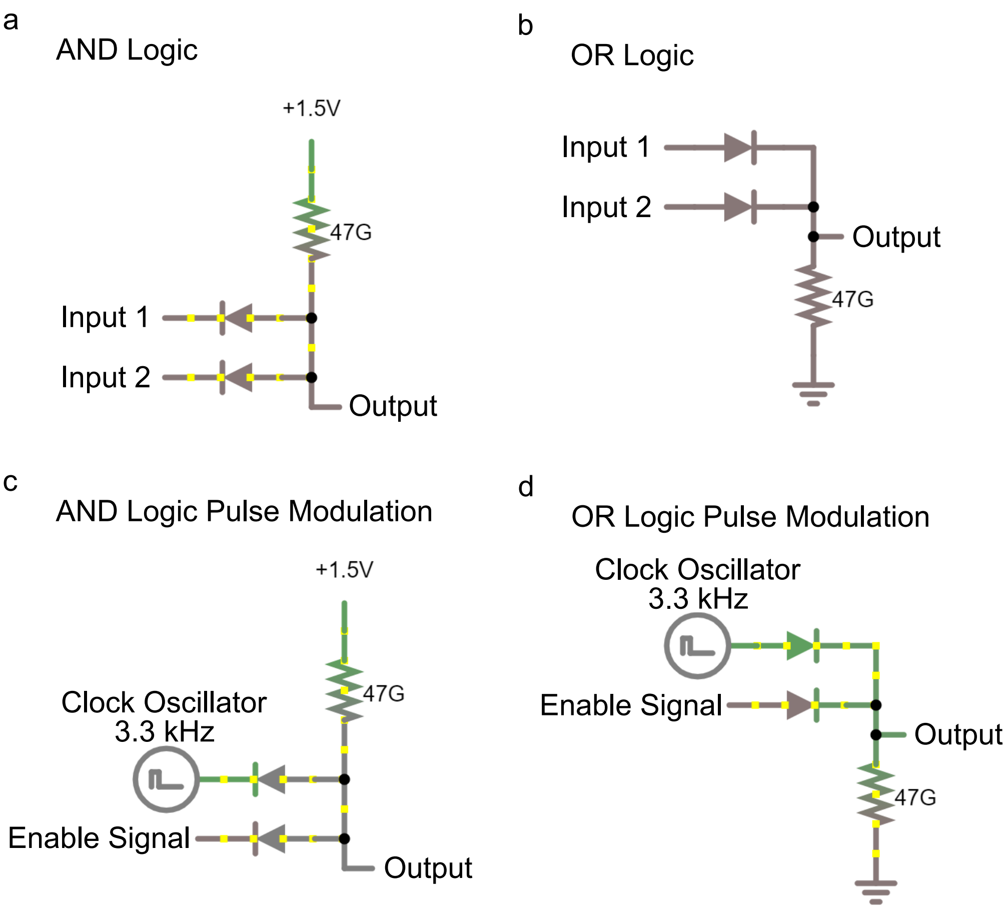

Supplementary Figure 20. Schematics of the simulated logic circuits comprising Au^{mica}/PCBA//PSI//EGaIn junctions and Au^{mica}/PCBM//PSI//EGaIn junctions using CircuitJS1. a, Schematic of the simulated AND logic circuit versus varied input under a constant load of 1.5 V. b, Schematic of the simulated OR logic circuit. c, Schematic of the simulated pulse modulation achieved by two AND logic circuits. d, Schematic of the simulated pulse modulation achieved by two OR logic circuits. Diodes are set with a saturation current of 3 pA, a series resistance of 1.5 GΩ, an emission coefficient of 2. Resistors are set with a resistance of 47.4 GΩ. Clock oscillators are set with a maximum voltage of 1.5 V in the waveform of pulse with a frequency of 3.3 kHz.

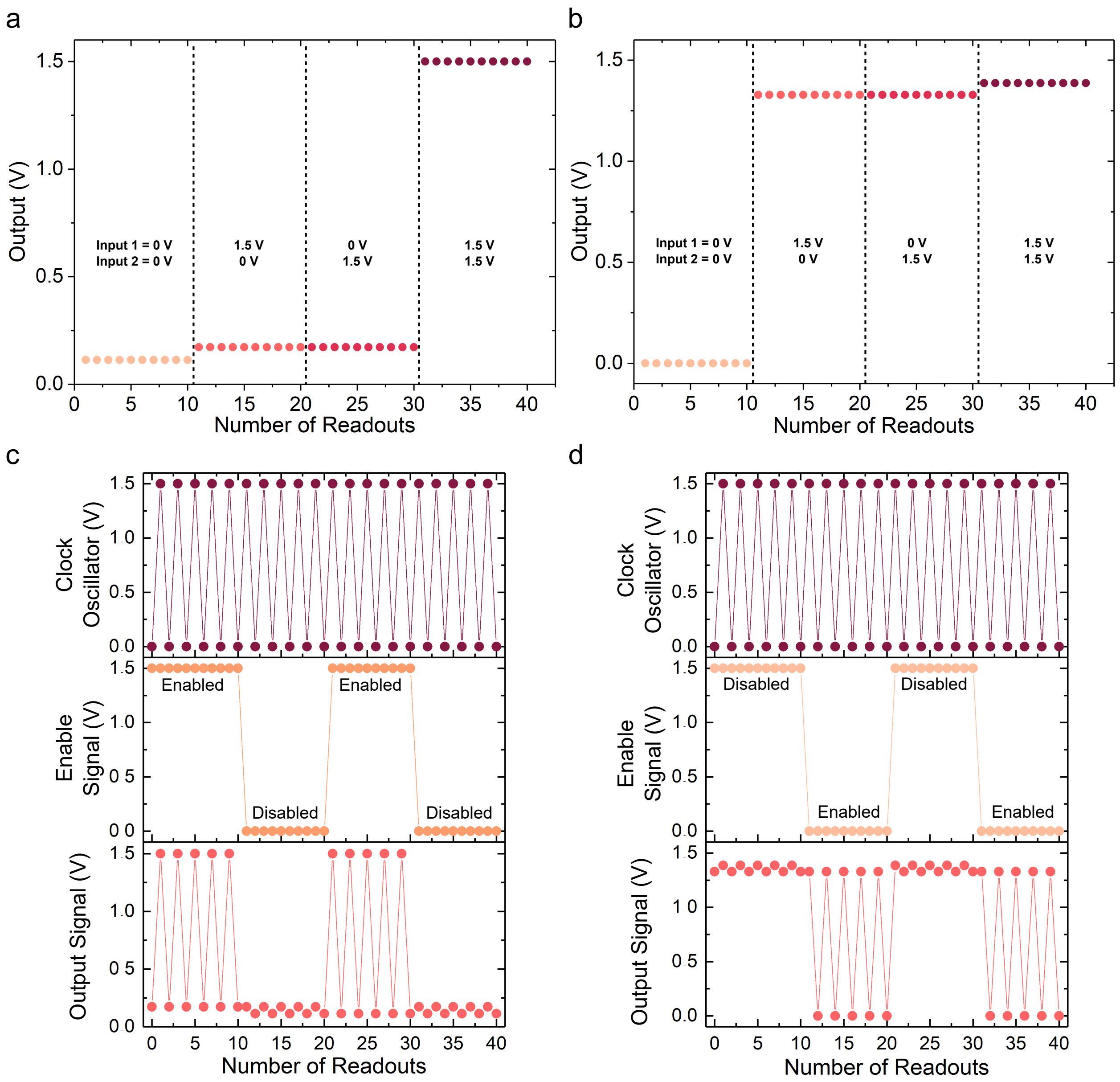

Supplementary Figure 21. Simulation of the functions of logic circuits comprising Au^{mica}/PCBA//PSI//EGaIn junctions and Au^{mica}/PCBM//PSI//EGaIn junctions. a, Plot of simulated output voltage of AND logic circuits versus varied input under a constant load of 1.5 V. b, Plot of simulated output voltage of OR logic circuits versus varied input. c, Simulated pulse modulation achieved by two AND logic circuits. d, Simulated pulse modulation achieved by two OR logic circuits. The specifications of each simulated electrical component are reproduced from their actual experimental performance. Diodes are set with a saturation current of 3 pA, a series resistance of 1.5 GΩ, an emission coefficient of 2. Resistors are set with a resistance of 47.4 GΩ. Clock oscillators are set with a maximum voltage of 1.5 V in the waveform of pulse with a frequency of 3.3 kHz.

5 Determination of thicknesses and PSI orientation

Our previous study on the self-assembled bilayers of fullerene derivatives with X-ray reflectivity confirmed that C_{60} cages as anchoring groups contribute 0.9 nm to the thickness;[5] the distance from the bottom of C_{60} fullerene to the carboxylic group of a PCBA molecule with minimized energy is 1.1 nm. The high yields of both EGaIn and CP-AFM junctions comprising SAMs of PCBA showed that the molecules are densely packed on the substrate. We therefore estimated the thickness of the SAMs of PCBA to be approximately 1 nm.

The structure of PSI trimers has been studied extensively by high-resolution X-ray techniques;[6–8] PSI trimer has a diameter of 21 nm and a maximal height of 9 nm. Because of its relatively large aspect ratio (i.e., the ratio between its height and diameter), height analysis by tapping mode AFM can provide evidence for the orientation of PSI trimers, e.g., side-oriented PSI trimers (the natural direction of electron flow inside the protein) is parallel to the substrate) is significantly taller than up-/down-oriented ones (the natural direction of electron flow is against/toward the substrate), and the formation of SAMs (e.g., aggregates of PSI trimers are beyond the dimensions of an individual protein). The combined results from height analysis and electrical characterization can effectively determine the exact orientation of PSI trimers, as demonstrated in our previous work..[4,9] In this work, height analysis on the SAMs of PSI on PCBA with a Gaussian fit yields a statistical mean of 8.93 nm and a full width at half maximum (FWHM) of 0.55 nm (Supplementary Figure 22), suggesting the formation of monolayers of PSI trimers and that the complexes form a 9 nm physical barrier in tunneling junctions.

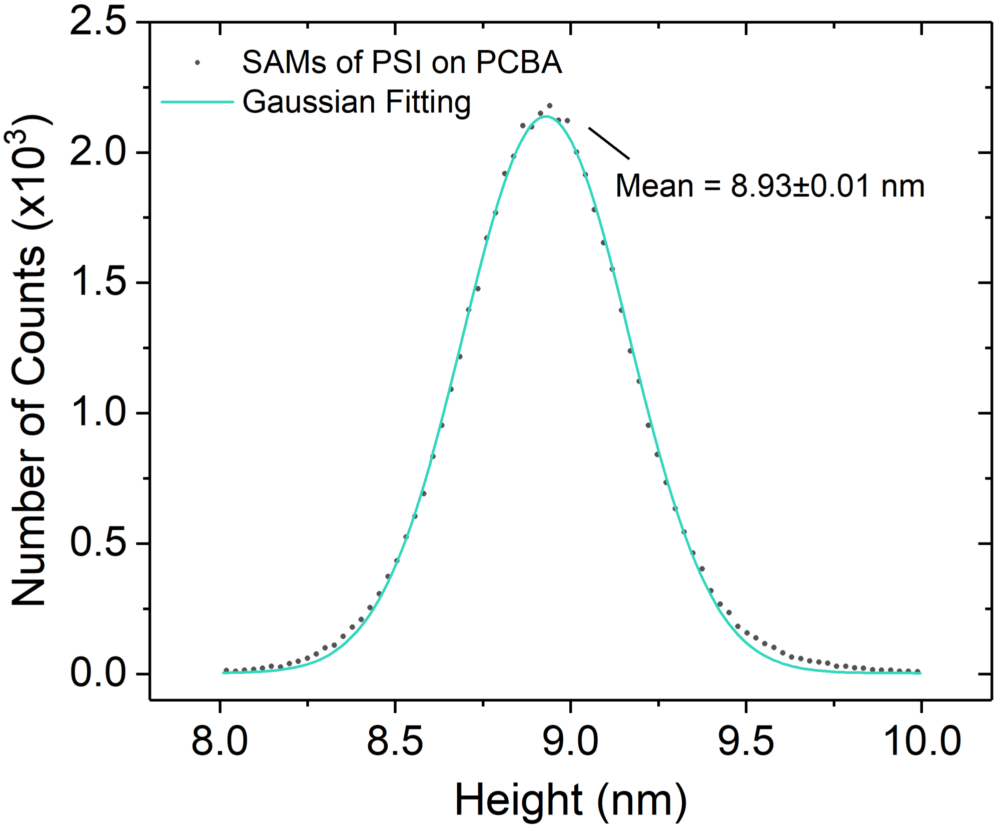

Supplementary Figure 22. Distribution of step heights measured on the SAMs of PSI on PCBA using tapping mode AFM. A Gaussian fitting to the distribution produces a statistical mean of 8.93 nm, suggesting the formation of SAMs.

We further verified the orientation of PSI from the statistical distribution of rectification ratios of individual PSI measured in CP-AFM junctions. As shown in Supplementary Figure 23a, over 98% of the junctions comprising SAMs of PSI on PCBA showed rectification of electrical current, resulting in a Gaussian mean of 2.76 at 2 V. In contrast, junctions comprising SAMs of PSI on PCBM showed lower rectification ratios in Supplementary Figure 23b, resulting in a Gaussian mean at 0.33 and broader distribution (i.e., a standard deviation of 0.21 in contrast to 0.06 from the SAMs of PSI on PCBA). We have previously established that the alignment between the built-in

dipole of PSI facilitated by its orientation and the external electric field gives rise to rectification of tunneling current across the junction (Ref. 4 and 9, and a discussion on rectification mechanism in this manuscript); we therefore concluded that 98 % of the PSI on PCBA linkers were oriented down.

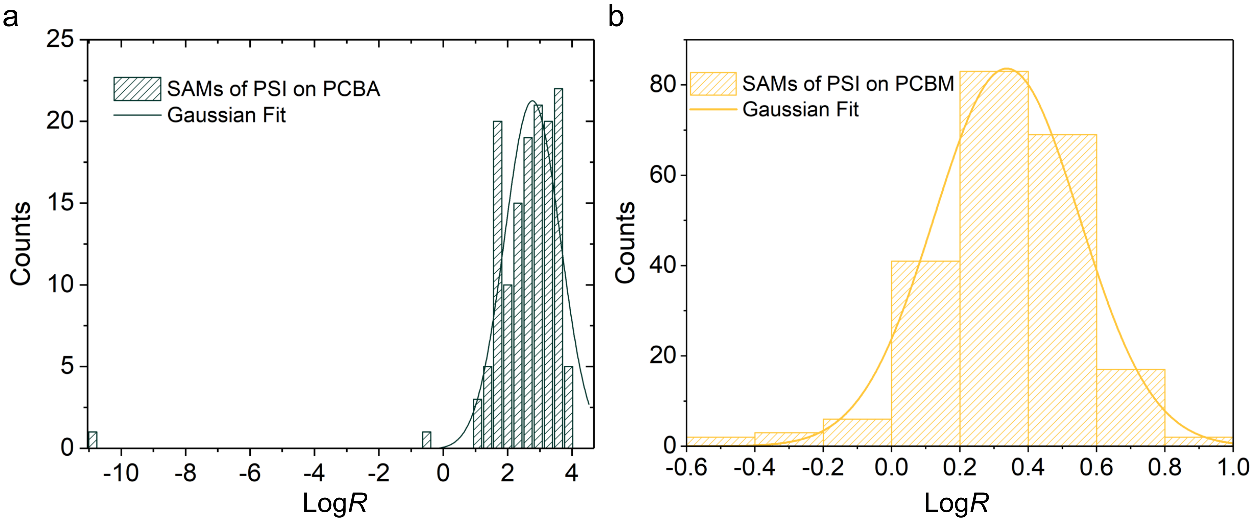

Supplementary Figure 23. Histograms of log$R$ measured in Au^{mica}/PCBA//PSI//Au^{AFM} (a) and Au^{mica}/PCBM//PSI//Au^{AFM} (b) junctions. a, Histograms of log$R$ measured at 2 V in Au^{mica}/PCBA//PSI//Au^{AFM} junctions are fitted to a Gaussian distribution to produced a Gaussian mean of 2.76 with a standard deviation of 0.06. b, Histograms of log$R$ measured at 2 V in Au^{mica}/PCBM//PSI//Au^{AFM} junctions are fitted to a Gaussian distribution to produced a Gaussian mean of 0.33 with a standard deviation of 0.33.

6 Possible models for charge-transport mechanisms

Here, we considered charge-transport in the inverted Marcus region, via voltage-induced long-range coherent electron transfer, via flickering resonance, proton-coupled electron transfer for the charge-transport mechanism in PSI junctions.

Charge-transfer in PSI that contributes to the charge recombination may operate in the inverted Marcus region to result in high internal quantum efficiency, i.e., charge recombination between the oxidized P700 cofactor and the reduced A1 cofactor is inhibited;[10] however, excitons were not present as the junctions comprising PSI SAMs in this work were characterized in the dark, and variable temperature measurements of the junctions showed that a thermally-activated charge-transfer necessary to generate a $P700^{+}A1^{-}$ pair is absent. Therefore we do not believe that charge transfer in the inverted Marcus region is responsible for the asymmetric charge-transport across PSI junctions.

In the model of voltage-induced long-range coherent electron transfer developed by Naaman and coworkers, electronic states in the molecule are strongly coupled to their neighboring sites so that the density of states is nearly continuous to mediate temperature-independent resonant tunneling.[11] In this model, the current density of the junction in logarithmic scale (e.g., $\mathrm{Ln}|J|$) is proportional to the reciprocal of applied bias (i.e., V^{-1}), which is not found the PSI junctions as shown in Supplementary Figure 24. The model might be able to explain the charge-transport across PSI junctions at the high-bias region where linearity is observed, but not the non-linear transition to low-bias region and the origin of rectification.

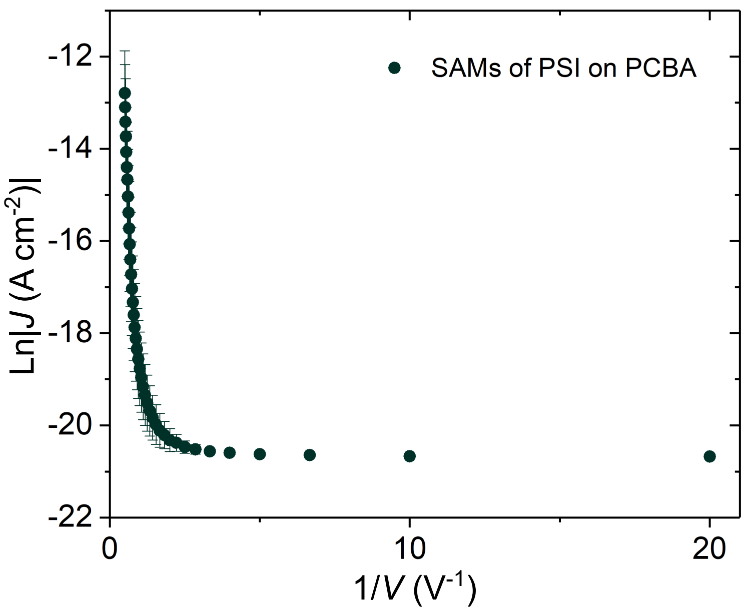

Supplementary Figure 24. Plots of Ln$|J|$ versus the reciprocal of potential in Au^{mica}/PCBA//PSI//EGaIn junctions from 0 V to 2 V. Error bars represent standard deviations.

The flickering resonance model, developed by Beratan and coworkers, rationalizes that charge-transport over long distance in macromolecules can be mediated by the energy states generated from the fluctuations of the molecular structure and the medium, which come into resonance with the Fermi levels of the electrodes.[12] Although the model provides a good approximation on the exponential decay of current through macromolecules, the prerequisite fluctuations on the donor and acceptor unites in the molecules is temperature-dependent, which is not found in the variable temperature measurements of the PSI junctions. We therefore do not believe that the flickering resonance model applies to the charge-transport across PSI junctions.

Cahen and coworkers observed the transition from off-resonant to on-resonant tunneling across junctions comprising SAMs of metalloproteins, in which the spacer molecule modifies the

coupling between the protein and the electrode to allows the states of cofactors containing Cu(II) ions to mediate efficient charge-transport.[13] In PSI trimers, the subunits containing metal ions (e.g., the P700 cofactor contains Mg cations and the F_x cofactor contains Fe cations) facilitate efficient charge-transfer via the electron transport chain by hopping; since the polarity of rectification of the PSI junctions opposes the redox gradient of the electron transport chain as shown in Supplementary Figure 9, the subunits do not seem to facilitate the asymmetric charge-transport across the PSI junctions. Both SAMs of PSI on PCBA and PCBM, despite the different orientation of PSI that may lead to varied protein-electrode coupling, facilitate efficient charge-transport with a tunneling decay coefficient smaller than 0.2 $Å^{-1}$, which suggests that long-range coherent tunneling is a property of folded polypeptides and not metalloproteins.

Proton-coupled electron transfer (PCET) describes the concerted (simultaneous) or sequential transfer of strongly-interacting protons and electrons from one atom to another, creating an energetically-favored pathway over decoupled electron and proton transfers in biological processes such as photosynthesis.[14,15] It can facilitate long-range and/or efficient charge transfer in proteins, e.g., proton-coupled electron transfers over 3-4 nm were observed on [FeFe]-hydrogenases,[16] and a near-zero tunneling decay coefficient for proton transfer was found on phenol-amines.[17] In contrast to solid-state large-area junctions comprising SAMs, molecular systems that exhibit PCET are usually characterized in solutions with varied pH. Although low activation energies were reported in several molecular systems that facilitate PCET, they unambiguously showed temperature-dependence on rate of proton/electron transfer which can be ascribed to i) the Boltzmann population of reactant proton vibrational states, ii) the thermal distribution of proton tunneling distances, and

iii) the classical barriers for the vibronic transitions.[18,19] We cannot probe PCET through PSI SAMs using our experimental setups, but we discussed it here as a possible mechanism of charge-transport.

Whitesides and coworkers reported in a series of works that tunneling charge-transport is insensitive to the polarity of functional groups in thiol-SAMs,[20–22] in particular, the change in molecular dipole yields similar conductance in the SAMs and rectification ratios close to 1 at low biases (from −0.5 V to 0.5 V). These findings, together with the work by Baghbanzadeh and coworkers,[23,24] established that oligopeptides can mediate efficient tunneling charge-transport and the molecular dipole does not facilitate asymmetric charge-transport. These findings are not in conflict with our work. The dipoles that generate the electric field in PSI are not from individual peptides or polar groups, they are primarily the result of the alpha helices that surround the reaction center, which collectively exert a significantly larger influence than that of an oligopeptide or polar group. The reaction center itself is also polarized. Thus, it is the precise orientation of the dipoles within each PSI complex that gives rise to rectification, which is why we are able to observe it in single-complexes by CP-AFM, but to observe it in large-area junctions, most/all of the complexes must be oriented in the same direction so as not to cancel out.[4] We have previously observed vacuum level shifts induced by molecular dipoles in SAMs[25] that affect tunneling charge-transport[26] enough to induce rectification.[27] Thus, we are confident that sufficiently large (collective) dipole moments can affect charge-transport characteristics including rectification, and recognize the importance of contextualizing our observations with prior work to underscore that it is not as straightforward as simply installing dipole moments in a SAM and observing rectification. That distinction also supports our hypothesis that it is the loss of such alignment that eliminates rectification, while retaining

efficient charge-transport, as shown by the control experiments (e.g., comparison between PCBA and PCBM linkers, and comparisons between PSI, BSA and denatured PSI).

There are examples of coherent tunneling transport further than 9 nm in molecular ensembles. Slinker and coworkers reported effective coherent tunneling charge-transport across the SAMs of thiol-functionalized double-stranded DNAs with 100 and 17 base pairs on Au electrode, which resulted in a tunneling decay coefficient of 0.05 $Å^{-1}$ over 34 nm.[28] In that work, the charge-transport properties of the SAMs were characterized by measuring the rate of charge transfer between the terminal Nile Blue redox probe and the Au electrode by cyclic voltammetry, in which the spatial separation of the probe and the electrode is defined by the length of DNA. Slinker and coworkers rationalized that the delocalized domains of the π-stacked bases formed band-like structures that mediate efficient charge-transport. Saxena and coworkers reported coherent tunneling charge-transport across 5-20 nm in carbon-based large-area junctions comprising electrochemically grafted monolayers of nitroazobenznenes.[29] In this work, the lowest unoccupied molecular orbitals of the oligonitroazobenzenes were drawn to near-resonance by the electric field to facilitate activationless (or temperature-independent) coherent tunneling charge-transport at high bias.

7 Analysis on skewness and kurtosis

We applied the analysis of skewness and kurtosis on the distribution of currents to EGaIn junctions comprising the SAMs of dodecanethiols (C12SH), PCBA, PSI on PCBM and PSI on PCBA,

and plotted the evolution of statistical moments against bias (-0.5 V to 0.5 V for the SAMs of C12SH; -1 V to 1 V for the SAMs of PCBA; -2 V to 2 V for the SAMs of PSI on PCBM/PCBA) in Supplementary Figure 25. The skewness (Supplementary Figure 25a for positive bias, and b for negative bias) and kurtosis (Supplementary Figure 25c for positive bias, and d for negative bias) of the reference SAMs of C12SH were independent of the applied bias, which is in agreement with the observation by Chen and coworkers.[30] Apart from their similar independent of bias, we observed larger skewness and kurtosis in the SAMs of C12SH (i.e., 3.7 for skewness and 13 for kurtosis) compared to literature (i.e., -0.80 for skewness and 0.50 for kurtosis). The skewness of the SAMs of PCBA fluctuated around 0 (Supplementary Figure 25a and b), while the kurtosis gradually increased (from -0.35 to 0.13 at positive bias, and from -0.37 to 0.76 at negative bias) with increasing bias (Supplementary Figure 25c and d), suggesting a convergence of resistance. At positive bias, the skewness of the SAMs of PSI on PCBA and PCBM increased (from -0.41 to 0.64 for PSI/PCBA, and from -0.84 to 1.6 for PSI/PCBM) until 0.9 V and 1.2 V, then it stabilized around 0.48 for PSI/PCBA but gradually decreased to 0.42 for PSI/PCBM at 2 V, respectively (Supplementary Figure 25a); similarly, the kurtosis of the SAMs of PSI on PCBA and PCBM increased (from -0.65 to 0.36 for PSI/PCBA, and from 0.83 to 2.8 for PSI/PCBM) until 0.9 V and 1.2 V, then the former fluctuated around 0 and the latter decreased to -0.17, respectively (Supplementary Figure 25c). At negative bias, the skewness and kurtosis of both SAMs fluctuated with increasing bias (Supplementary Figure 25b and d), though we noticed that the skewness of the SAMs of PSI on PCBA and PCBM stabilized at 1.1 and 0 at higher biases (e.g., from -1.6 V to -2 V). The transition from negatively-skewed distribution to positively-skewed distribution observed in the SAMs

of PSI on PCBA at low positive bias suggests the emergence of high-conductance tails with increasing bias and the alignment of molecular dipole and external field; the decrease in skewness in the SAMs of PSI on PCBM at high positive bias suggests the disappearance of high-conductance tail, and may correspond to the misalignment of molecular dipole and external field as the latter becomes dominant, in contrast to the more aligned SAMs of PSI on PCBA. The SAMs of PSI on PCBM showed a stronger bias-dependence of kurtosis than the SAMs of PSI on PCBA, with the convergence and divergence of conductance before and after 1.2 V. However, we cannot interpret the fluctuation in the skewness and kurtosis of the SAMs of PSI on PCBA and PCBM, since we cannot verify if it originated from the angular orientation of the dipole moment due to the complexity of PSI, particularly in bilayers with fullerene derivatives. Chen and coworkers applied this analysis to a much better-defined system in which the orientation of the dipoles with respect to the surface could be assumed with a reasonable degree of certainty. Regardless, the skewness and kurtosis of our J/V data are consistent with a mechanism of charge-transport in which oriented dipoles partially mitigate conductance, further supporting the hypothesis that it is the (lack of) oriented dipoles that leads to (the lack of) rectification when PSI is (randomly) oriented by director SAMs of fullerenes.

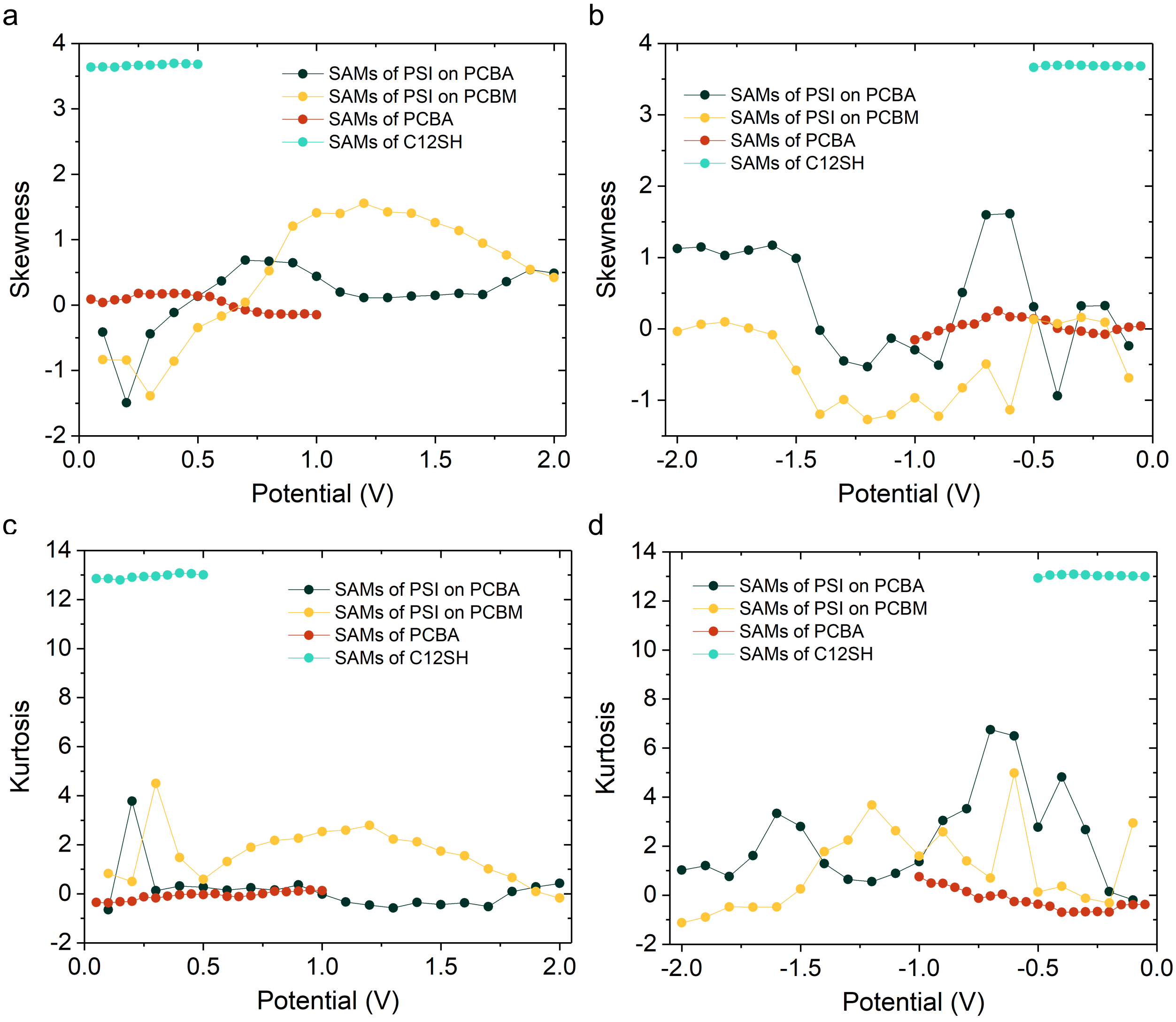

Supplementary Figure 25. Plots of skewness (a and b) and kurtosis (c and d) versus potential (positive bias in a and c, negative in b and d) in Au^{TS}/C12SH//EGaIn junctions from −0.5 V to 0.5 V, Au^{mica}/PCBA//EGaIn junctions from −1 V to 1 V, Au^{mica}/PCBM//PSI//EGaIn junctions and Au^{mica}/PCBA//PSI//EGaIn junctions from −2 V to 2 V.

To understand the applicability of the analysis on rectifying molecular junctions (in contrast, Chen and coworkers reported junctions that exhibited symmetric charge-transport), we investigated the bias-dependence of skewness and kurtosis in the SAMs of ferrocenyl undecanethiols (FcC11SH, the embedded dipole is directed along the backbone of the molecule) and the self-assembled bilay-

ers (SABs) of triethylene glycol-functionalized fullerenes (PTEG-1, forming symmetric molecular self-assemblies in which the interdigitated TEG chains are sandwiched by fullerenes at the top and bottom interfaces) using EGaIn junctions, as shown in Supplementary Figure 26. Asymmetric charge-transport through both self-assemblies is facilitated by thermally-activated processes, in particular, hopping mediated by molecular frontier orbitals;[31–33] however, their internal dipoles are vastly different due to their structures (i.e., FcC11SH possesses a built-in dipole from ferrocene to sulfur, while the molecular dipole is canceled out in the SABs of PTEG-1). To disentangle the contribution from hopping, we plotted the evolution of skewness and kurtosis of both self-assemblies in the bias windows where charge-transport is via direct tunneling (i.e., 0 V to 1 V for SAMs of FcC11SH in Supplementary Figure 26a, and −1.5 V to 0 V for SABs of PTEG-1 in Supplementary Figure 26b). The skewness of the SAMs of FcC11SH fluctuated from 0.066 to 0.87 and the kurtosis fluctuated from -1.1 to 1.5; at low bias (e.g., <0.5 V), the skewness decreased from 0.38 to 0.22 and the kurtosis decreased from 0.70 to -0.72 with increasing bias. The skewness of the SABs of PTEG-1 decreased from 0.080 to -0.19 as the bias decreased from 0 V to −0.3 V, then increased to 0.13 at −0.9 V, before gradually decreasing again to 0.054 at −2 V; the kurtosis decreased from 0.066 to -0.62 as the bias changed from 0 V to −0.5 V despite a spike at −0.3 V, before gradually increasing to -0.51 at −2 V. The SAMs of FcC11SH and the SABs of PTEG-1 both showed bias-dependence on skewness and kurtosis despite their difference in dipoles.

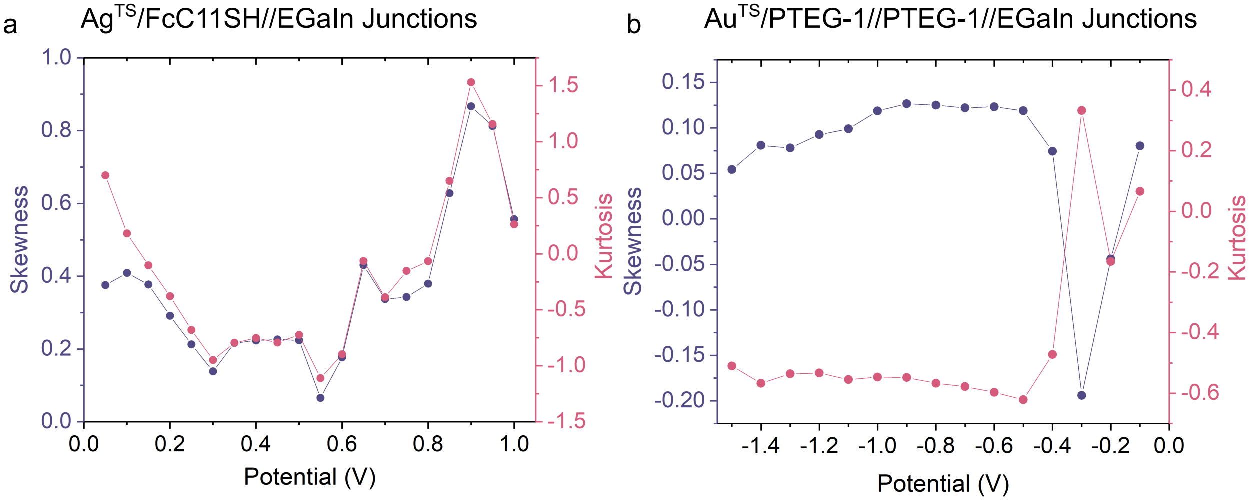

Supplementary Figure 26. Plots of skewness and kurtosis versus potential in Ag^{TS}/FcC11SH//EGaIn junctions from 0 V to 1 V (a) and Au^{TS}/PTEG-1//PTEG-1//EGaIn junctions from −1.5 V to 0 V (b).

In summary, we found the bias-dependence of skewness and kurtosis in the SAMs of PSI on PCBA, in which asymmetric charge-transport is mediated by the alignment of internal dipole and external field, and in the SAMs of FcC11SH and the SABs of PTEG-1, in which asymmetric charge-transport is dominated by tunneling-hopping mechanism. Though we observed unidirectional changes of these statistical moments at low bias, such patterns were lost for all investigated systems at an extended bias window. The work by Chen and coworkers showed the bias-dependence of skewness and kurtosis in symmetric charge-transport at low bias, but lacked insights into asymmetric charge-transport in large-area junctions over an extended range of bias. We therefore cannot correlate the skewness and kurtosis extrapolated from the molecular junctions reported in our work to the change of intermolecular dipole under external electric field.

Supplementary References

(1) Hummelen, J. C.; Knight, B. W.; Lepeq, F.; Wudl, F.; Yao, J.; Wilkins, C. L. *J. Org. Chem.* **1995**, *60*, 532–538.

(2) Jahani, F.; Torabi, S.; Chiechi, R. C.; Koster, L. J. A.; Hummelen, J. C. *Chem. Commun.* **2014**, *50*, 10645–10647.

(3) Weiss, E. A.; Kaufman, G. K.; Kriebel, J. K.; Li, Z.; Schalek, R.; Whitesides, G. M. *Langmuir* **2007**, *23*, 9686–9694.

(4) Ocampo, O. E. C.; Gordiichuk, P.; Catarci, S.; Gautier, D. A.; Herrmann, A.; Chiechi, R. C. *J. Am. Chem. Soc* **2015**, *137*, 8419–8427.

(5) Qiu, X.; Ivasyshyn, V.; Qiu, L.; Enache, M.; Dong, J.; Rousseva, S.; Portale, G.; Stöhr, M.; Hummelen, J. C.; Chiechi, R. C. *Nat. Mater.* **2020**, 330–337.

(6) Hladík, J.; Sofrová, D. *Photosynth. Res.* **1991**, *29*, 171–175.

(7) Krauss, N.; Hinrichs, W.; Witt, I.; Fromme, P.; Pritzkow, W.; Dauter, Z.; Betzel, C.; Wilson, K. S.; Witt, H. T.; Saenger, W. *Nature* **1993**, *361*, 326–331.

(8) Fromme, P.; Jordan, P.; KrauSS, N. *Biochim. Biophys. Acta* **2001**, *1507*, 5–31.

(9) Gordiichuk, P.; Pesce, D.; Ocampo, O. E. C.; Marcozzi, A.; Wetzelaer, G.-J. A. H.; Paul, A.; Loznik, M.; Gloukhikh, E.; Richter, S.; Chiechi, R. C.; Herrmann, A. *Adv. Sci.* **2017**, *4*, 1600393.

(10) Makita, H.; Hastings, G. *PNAS* **2017**, *114*, 9267–9272.

(11) Michaeli, K.; Beratan, D. N.; Waldeck, D. H.; Naaman, R. *PNAS* **2019**, *116*, 5931–5936.

(12) Zhang, Y.; Liu, C.; Balaeff, A.; Skourtis, S. S.; Beratan, D. N. *PNAS* **2014**, *111*, 10049–10054.

(13) Fereiro, J. A.; Yu, X.; Pecht, I.; Sheves, M.; Carlos, J.; Cahen, D. *PNAS* **2018**, *115*, E4577–E4583.

(14) Hammes-Schiffer, S.; Soudackov, A. V. *J. Phys. Chem. B* **2008**, *112*, 14108–14123.

(15) Tyburski, R.; Liu, T.; Glover, S. D.; Hammarström, L. *J. Am. Chem. Soc.* **2021**, *143*, 560–576.

(16) Lampret, O.; Duan, J.; Hofmann, E.; Winkler, M.; Armstrong, F. A.; Happe, T. *PNAS* **2020**, *117*, 20520–20529.

(17) Markle, T. F.; Rhile, I. J.; Mayer, J. M. *J. Am. Chem. Soc.* **2011**, *133*, 17341–17352.

(18) Rhile, I. J.; Markle, T. F.; Nagao, H.; DiPasquale, A. G.; Lam, O. P.; Lockwood, M. A.; Rotter, K.; Mayer, J. M. *J. Am. Chem. Soc.* **2006**, *128*, 6075–6088.

(19) Knapp, M. J.; Rickert, K.; Klinman, J. P. *J. Am. Chem. Soc.* **2002**, *124*, 3865–3874.

(20) Yoon, H. J.; Shapiro, N. D.; Park, K. M.; Thuo, M. M.; Soh, S.; Whitesides, G. M. *Angew. Chem.* **2012**, *124*, 4736–4739.

(21) Thuo, M. M.; Reus, W. F.; Simeone, F. C.; Kim, C.; Schulz, M. D.; Yoon, H. J.; Whitesides, G. M. *J. Am. Chem. Soc.* **2012**, *134*, 10876–10884.

(22) Yoon, H. J.; Bowers, C. M.; Baghbanzadeh, M.; Whitesides, G. M. *J. Am. Chem. Soc.* **2014**, *136*, 16–19.

(23) Baghbanzadeh, M.; Bowers, C. M.; Rappoport, D.; aba, T.; Gonidec, M.; Al-Sayah, M. H.; Cyganik, P.; Aspuru-Guzik, A.; Whitesides, G. M. *Angew. Chem. Int. Ed.* **2015**, *54*, 14743–14747.

(24) Baghbanzadeh, M.; Belding, L.; Yuan, L.; Park, J.; Al-Sayah, M. H.; Bowers, C. M.; Whitesides, G. M. *J. Am. Chem. Soc.* **2019**, *141*, 8969–8980.

(25) Abu-Husein, T.; Schuster, S.; Egger, D. A.; Kind, M.; Santowski, T.; Wiesner, A.; Chiechi, R.; Zojer, E.; Terfort, A.; Zharnikov, M. *Adv. Funct. Mater.* **2015**, *25*, 3943–3957.

(26) Kovalchuk, A.; Abu-Husein, T.; Fracasso, D.; Egger, D. A.; Zojer, E.; Zharnikov, M.; Terfort, A.; Chiechi, R. C. *Chem. Sci.* **2016**, *7*, 781–787.

(27) Kovalchuk, A.; Egger, D. A.; Abu-Husein, T.; Zojer, E.; Terfort, A.; Chiechi, R. C. *RSC Adv.* **2016**, *6*, 69479–69483.

(28) Slinker, J. D.; Muren, N. B.; Renfrew, S. E.; Barton, J. K. *Nat. Chem.* **2011**, *3*, 228–233.

(29) Saxena, S. K.; Tefashe, U. M.; McCreery, R. L. *J. Am. Chem. Soc.* **2020**, *142*, 15420–15430.

(30) Chen, J.; Kim, M.; Gathiaka, S.; Cho, S. J.; Kundu, S.; Yoon, H. J.; Thuo, M. M. *J. Phys. Chem. Lett.* **2018**, *9*, 5078–5085.

(31) Nijhuis, C. A.; Reus, W. F.; Barber, J. R.; Dickey, M. D.; Whitesides, G. M. *Nano Lett.* **2010**, *10*, 3611–3619.

(32) Yuan, L.; Wang, L.; Garrigues, A. R.; Jiang, L.; Annadata, H. V.; Antonana, M. A.; Barco, E.; Nijhuis, C. A. *Nat. Nanotechnol.* **2018**, *13*, 322–329.

(33) Qiu, X.; Rousseva, S.; Ye, G.; Hummelen, J. C.; Chiechi, R. C. *Adv. Mater.* **2020**, *33*, 2006109.
